# Modeling cross-regulatory influences on monolignol transcripts and proteins under single and combinatorial gene knockdowns in *Populus trichocarpa*

**DOI:** 10.1371/journal.pcbi.1007197

**Published:** 2020-04-10

**Authors:** Megan L. Matthews, Jack P. Wang, Ronald Sederoff, Vincent L. Chiang, Cranos M. Williams

**Affiliations:** 1 Department of Electrical and Computer Engineering, North Carolina State University, Raleigh, North Carolina, United States of America; 2 State Key Laboratory of Tree Genetics and Breeding, Northeast Forestry University, Harbin, China; 3 Department of Forestry and Environmental Resources, Forest Biotechnology Group, North Carolina State University, Raleigh, North Carolina, United States of America; 4 Department of Forest Biomaterials, North Carolina State University, Raleigh, North Carolina, United States of America; University of Wisconsin, Madison, UNITED STATES

## Abstract

Accurate manipulation of metabolites in monolignol biosynthesis is a key step for controlling lignin content, structure, and other wood properties important to the bioenergy and biomaterial industries. A crucial component of this strategy is predicting how single and combinatorial knockdowns of monolignol specific gene transcripts influence the abundance of monolignol proteins, which are the driving mechanisms of monolignol biosynthesis. Computational models have been developed to estimate protein abundances from transcript perturbations of monolignol specific genes. The accuracy of these models, however, is hindered by their inability to capture indirect regulatory influences on other pathway genes. Here, we examine the manifestation of these indirect influences on transgenic transcript and protein abundances, identifying putative indirect regulatory influences that occur when one or more specific monolignol pathway genes are perturbed. We created a computational model using sparse maximum likelihood to estimate the resulting monolignol transcript and protein abundances in transgenic *Populus trichocarpa* based on targeted knockdowns of specific monolignol genes. Using *in-silico* simulations of this model and root mean square error, we showed that our model more accurately estimated transcript and protein abundances, in comparison to previous models, when individual and families of monolignol genes were perturbed. We leveraged insight from the inferred network structure obtained from our model to identify potential genes, including *PtrHCT*, *PtrCAD*, and *Ptr4CL*, involved in post-transcriptional and/or post-translational regulation. Our model provides a useful computational tool for exploring the cascaded impact of single and combinatorial modifications of monolignol specific genes on lignin and other wood properties.

## Author summary

Engineering trees to have desirable lignin and wood traits is of significant interest to the bioenergy and biomaterial industries. Genetically modifying the expression of the genes that drive the monolignol biosynthetic pathway is a useful method for obtaining new traits. Modifying the expression of one gene affects not only the abundance of its encoded protein, but can also indirectly impact the abundance of other transcripts and proteins. These proteins drive the monolignol biosynthetic pathway. Having an accurate representation of their abundances is key to understanding how lignin and wood traits are altered. We developed a computational model to estimate how the abundance of monolignol transcripts and proteins are changed when one or more monolignol genes are knocked down. Specifying only the abundances of the targeted genes as the input, our model estimates how the abundances of the untargeted transcripts and proteins are altered. Our model captures indirect regulatory influences at the transcript and protein levels observed in experimental data. The model is an important addition to current models of lignin biosynthesis. By incorporating our approach into the existing models, we expect to improve our ability to explore how new combinations of gene knockdowns impact lignin and many other wood properties.

## Introduction

Lignin is an important phenylpropanoid polymer that is embedded with cellulose and hemicelluloses in plant secondary cell walls [1, 2]. It plays an important role in plant physiology, defense, and adaptation by providing structural integrity, conducting water through vascular tissues, and acting as a barrier to pests and pathogens [1, 3]. Lignin is composed of three main sub-units, the *p*-hydroxyphenyl (H), guaiacyl (G), and syringyl (S) monolignols. These monolignols define the composition and interunit linkages that determine other characteristics of lignin [1, 2, 4]. How these monolignols are formed and synthesized into lignin has been an important research area for more than five decades [5].

**Fig 1:**
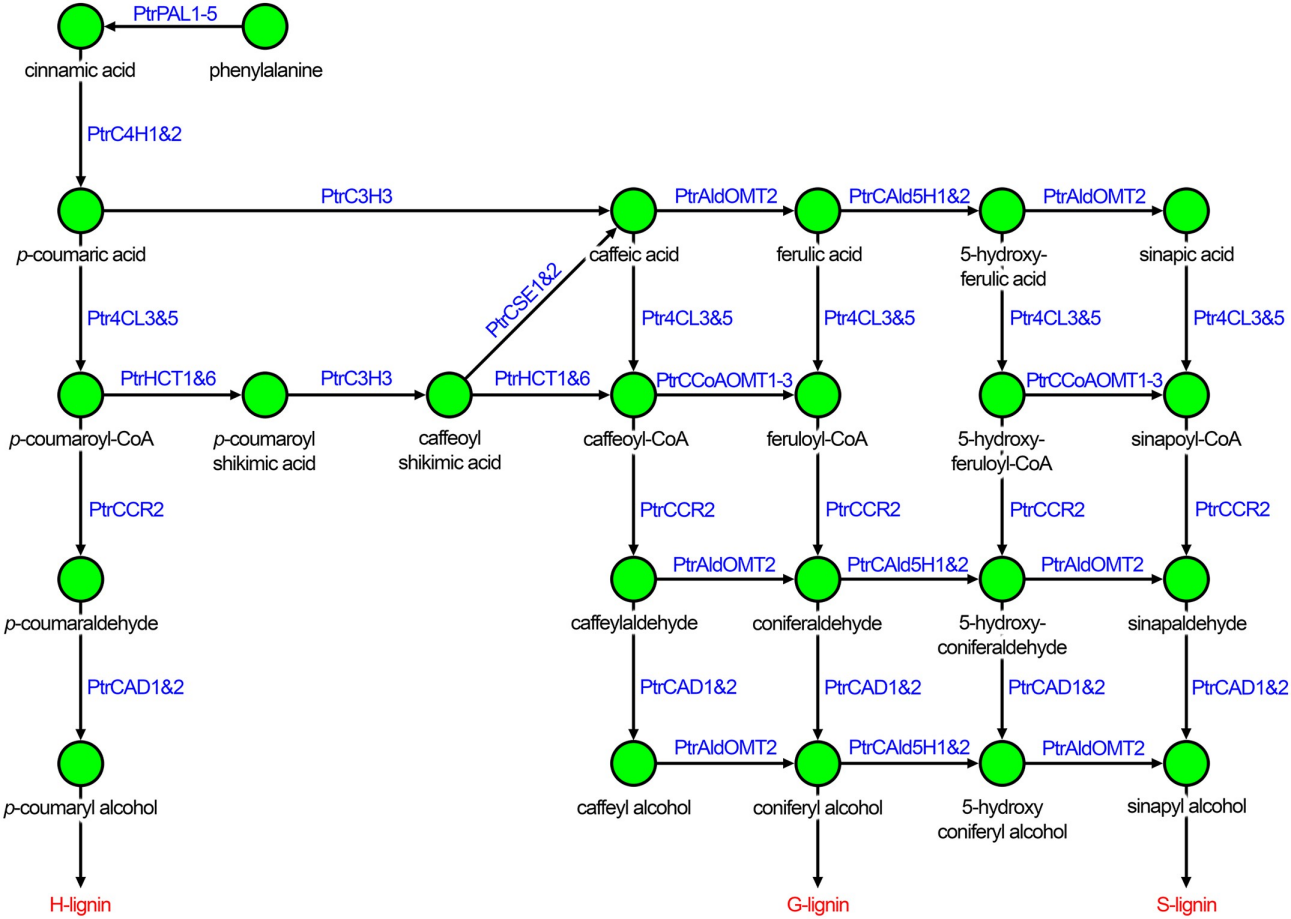
Monolignol biosynthetic pathway in *P. trichocarpa*.

The monolignol biosynthetic pathway is composed of a series of enzymatic reactions, involving 23 enzymes, that convert phenylalanine into the three monolignols through 24 intermediate metabolites (Fig 1). A key step to controlling lignin phenotypes is by precise manipulation of the monolignol biosynthesis pathway. Genetic modifications are a useful method for manipulating metabolic pathway behavior. These modifications alter transcript production or abundance resulting in a change to the amount of proteins available to catalyze key pathway reactions. It is not always intuitive how genetic modifications propagate through biological systems culminating in changes to phenotypic traits. Many approaches have been presented to understand phenotypic changes based on single layers of biological information, such as GWAS [6, 7] and QTL analysis [8, 9]. However, biological systems regulate themselves through diverse mechanisms including, transcriptional [10–12] and post-transcriptional [10, 13, 14] regulation, and post-translational modifications [14–16] among others. By improving our understanding of the factors that arise when knocking down genes, we can better discern how metabolic pathway activity and phenotypic responses change in response to knockdowns and other modifications.

Extensive study of the metabolic reactions associated with monolignol biosynthesis in *P. trichocarpa* has resulted in a detailed mechanistic computational model of the pathway, composed of 24 ordinary differential equations with 104 Michaelis-Menten and 103 inhibition kinetic parameters [17, 18]. Wang et al. expanded their mechanistic metabolic model of the monolignol pathway to incorporate information spanning the genome, transcriptome, proteome, and 25 lignin and wood traits [4]. This multi-scale model was used to help identify novel combinatorial genetic modifications that result in desired lignin and wood characteristics such as increased saccharification efficiency without negatively impacting plant growth. Wang et al., made the simplifying assumption that the abundance of each protein was dependent only on the transcript abundance of its monolignol gene. This simplification does not take into consideration potential epistatic transcriptional, post-transcriptional, or post-translational regulation mechanisms [12, 15, 16, 19–21] that may explain the sometime poor correlations between monolignol gene transcripts and proteins in some transgenic *P. trichocarpa* [4]. The development of approaches that can more accurately predict how the abundances of interconnected transcripts and proteins change under single and combinatorial transgenic knockdowns, while also providing insight on the topological structure of these cross-influences, would greatly improve our ability to predict how metabolic pathways and phenotypic traits will be altered under these modifications. In addition, these approaches would provide insight to the regulatory mechanisms responsible for the cross-influences among the transcripts and proteins. Such approaches would need to leverage available steady-state transcript and protein abundance data [4] but go beyond traditional inference and gene regulatory network modeling approaches such as Bayesian-based [22–24] or nested effect [25, 26] models to enable prediction of both transcript and protein abundances.

In this paper we performed differential abundance analyses on the monolignol gene transcript and protein abundances to further characterize epistatic influences on the expression of the monolignol genes in differentiating xylem tissue of *P. trichocarpa*. We then used the experimental transcript and protein abundance measurements [4] to develop a model that describes the indirect relationships between the monolignol genes as transcript to transcript, transcript to protein, protein to transcript, and protein to protein influences. To accomplish this, we adapted a modeling framework that is based on a structural equation framework that has been used to identify relationships between genes by incorporating eQTL information [27, 28]. Through the use of a sparse maximum likelihood (SML) estimator [28], our framework allows us to identify potential key indirect regulatory influences between the monolignol gene transcripts and proteins that we use to computationally estimate how the monolignol transcripts and protein abundances change in different transgenic simulations. Our model captures many of the putative epistatic influences between the monolignol transcripts and proteins by specifying only the abundances of the targeted transcripts as an input.

Through *in-silico* simulations, we show that our model more accurately estimates monolignol transcript and protein abundances in transgenic plants where individual and families of monolignol genes were knocked down than a model that does not incorporate such regulatory influences. We identified and modeled apparent regulatory influences among the *PtrCAld5H*, *Ptr4CL*, *PtrPAL*, *PtrC3H3*, *PtrC4H*, and *PtrHCT* gene families and among the *PtrHCT*, *Ptr4CL* gene families and *PtrCCoAOMT3*, which manifest as relationships between protein abundances but not the transcripts. Further, we identified two topological network motifs in our model that suggest the *PtrHCT*, *Ptr4CL*, and *PtrCAD* families are involved in the post-transcriptional or post-translational regulatory mechanisms, and would be good candidates for further experiments to identify the specific regulatory mechanisms responsible.

Predicting what transgenic modifications will lead to desired lignin and wood phenotypes is of current interest in the bioenergy and biomaterial industries among others [29, 30]. Computational models of the monolignol pathway have become an important tool in the past decade to understanding how changes to the monolignol enzymes result in changes to the pathway outputs [18, 31–34] and lignin and wood phenotypes [4]. We add to this body of work by developing a model that incorporates observed influences at both the transcript and protein levels to estimate how the enzymes in the monolignol biosynthetic pathway are influenced by one or more monolignol gene knockdowns.

## Results

### Data description

Wang et al. [4] performed a series of systematic transgenic experiments that knocked down 21 of the 23 lignin specific genes and their gene families in the model tree *P. trichocarpa*. The caffeoyl shikimate esterases (*PtrCSE1&2*) (Fig 1) were discovered after the onset of their study [4], and therefore were not included in their experiments or model. The absolute transcript abundances were measured using RNAseq, and the absolute protein abundances were obtained using protein cleavage coupled with isotope dilution mass spectrometry (PC-IDMS) [35]. Multiple independent lines were grown for each transgenic construct. Up to three of those lines were selected to show the effects of a range in the level of the targeted knockdown gene expression. This provided an indication of the complexity of putative interactions as responses can be linear or nonlinear. For each line, up to three biological replicates were collected after six months of growth, resulting in 207 transgenic measurement profiles and 18 wildtype measurement profiles. Due to limited greenhouse space, these experiments were grown in six batches. To account for batch effects on the data, Wang et al. normalized the data to the wildtype mean in each batch [4]. Additionally, the PC-IDMS approach for quantifying protein abundance was not able to differentiate between the *PtrPAL4* and *PtrPAL5* proteins because of the near identity of these proteins [35]. The transcript and protein abundances for *PtrPAL4* and *PtrPAL5* were combined into one, which we refer to as *PtrPAL4/5*.

### Differential abundance analysis

**Fig 2:**
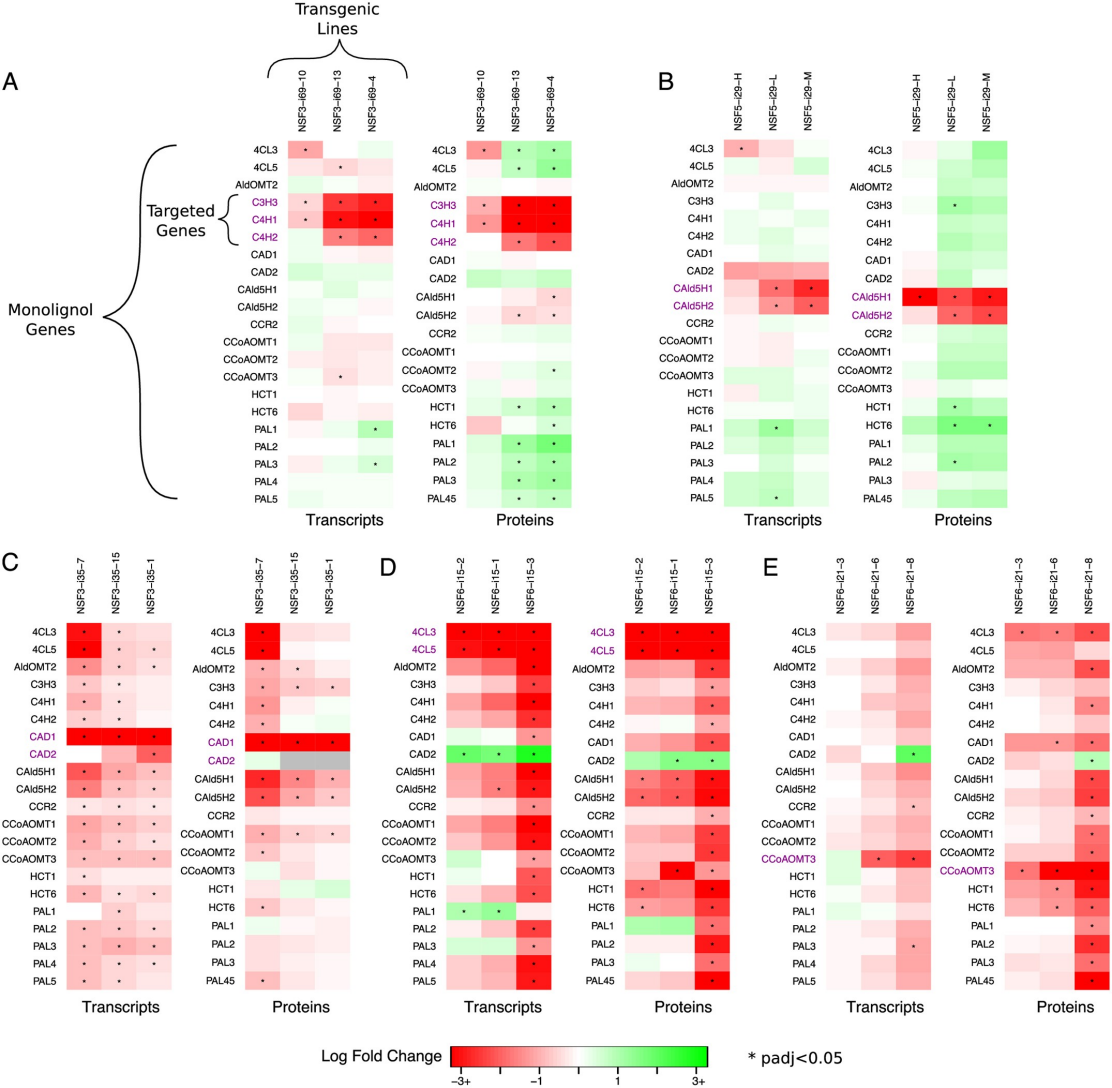
Monolignol gene transcript and protein differential abundance. (A) *PtrC3H3*, *PtrC4H1* and *PtrC4H2* knockdown experiments (Construct i69). (B) *PtrCAld5H1* and *PtrCAld5H2* knockdown experiments (Construct i29). (C) *PtrCAD1* and *PtrCAD2* knockdown experiments (Construct i35). (D) *Ptr4CL3* and *Ptr4CL5* knockdown experiments (Construct i15). (E) *PtrCCoAOMT3* knockdown experiments (Construct i21). Gray boxes are due to missing data. Rows are the monolignol gene names, with the targeted genes for each experiment in purple. Columns are the experimental lines. * indicates *p*_adj_<0.05.

To further examine the influence of targeted knockdowns on other non-targeted genes, we performed a differential abundance analysis on both the transcripts and protein data. Fig 2 contains heatmaps showing the results for five of the knockdown experiments: construct i69, which targeted *PtrC3H3*, *PtrC4H1*, and *PtrC4H2* (Fig 2A); construct i29, which targeted *PtrCAld5H1* and *PtrCAld5H2* (Fig 2B); construct i35, which targeted *PtrCAD1* and *PtrCAD2* (Fig 2C); construct i15, which targeted *Ptr4CL3* and *Ptr4CL5* (Fig 2D); and construct i21, which targeted *PtrCCoAOMT3* (Fig 2E). Heatmaps for the remaining transgenics can be found in S1–S4 Figs. Each column represents a different line of that experiment, with each line containing up to 3 replicates. The rows indicate the monolignol specific gene name with the purple names indicating the gene(s) that were knocked down. The colorscale of these heatmaps corresponds to the log fold change (logFC) from their wildtype. Red represents a negative fold change, i.e., a decrease in expression, and green corresponds to a positive fold change or an increase in expression. Gray boxes represent missing data. Changes in abundance that had a *p*-value adjusted for multiple comparisons less than 0.05 are considered statistically significant and are indicated with an asterisk.

We see significant changes in abundance in several of the untargeted monolignol genes. This indicates that there are cross-influences among the targeted monolignol genes impacting the abundances of untargeted monolignol transcripts and proteins. Collectively examining the responses of both the monolignol gene transcripts and proteins provides insight to the regulatory influences between the monolignol genes that would not be detected by just examining the transcripts. While we observe some instances of the same differential abundance patterns in the transcripts and proteins, suggesting transcriptional regulation, we also observe several cases where only a monolignol gene’s transcript or its protein abundance is significantly altered. This suggests the presence of post-transcriptional or post-translational regulation.

In the *PtrC3H3*, *PtrC4H1*, and *PtrC4H2* knockdown experiments we observe significant increases in the abundances of the *Ptr4CL*, *PtrHCT*, and *PtrPAL* proteins and significant decreases in the *PtrCAld5H* proteins (Fig 2A). However, their corresponding transcript abundances, with the exception of some of the *PtrPAL* transcripts, are not found to be differentially expressed. Similarly, in the *PtrCAld5H1* and *PtrCAld5H2* knockdown experiments we observe significant increases in the abundances of the *PtrHCT* and *PtrC3H3* proteins that are not observed in the transcript data (Fig 2B). In the *PtrCAD1* and *PtrCAD2* knockdown experiments (Fig 2C), we observe a decrease in the abundance of both the transcripts and proteins of *PtrCAld5H1* and *PtrCAld5H2*, as well as most of the other monolignol transcripts. Despite this, many of the proteins are not significantly different from their wildtype levels. This could be explained by the same behavior as in the *PtrCAld5H1* and *PtrCAld5H2* knockdowns and *PtrC3H3*, *PtrC4H1*, and *PtrC4H2* knockdown experiments where we also observed an increase in several of the protein abundances. The increase we observe in the proteins in those two knockdowns could lead to wildtype levels in the *PtrCAD1* and *PtrCAD2* knockdown experiments because the transcript abundances are significantly decreased. This behavior is seen to a lesser degree in the experimental line that had the largest decrease in the *Ptr4CL3* and *Ptr4CL5* transcripts and proteins. Additionally, we do not observe this behavior in the *Ptr4CL3* and *Ptr4CL5* knockdown experiments (Fig 2D), suggesting that large knockdowns of the *Ptr4CL* gene family may trump other regulatory influences.

In the *Ptr4CL3* and *Ptr4CL5* transgenics (Fig 2D), we observe significant decreases in abundance of both the transcripts and proteins of *PtrCAld5H1* and *PtrCAld5H2* and an increase in the *PtrCAD2* abundances across multiple transgenic lines. Significant decreases in abundance are also observed in the *PtrHCT1*, *PtrHCT6*, and *PtrCCoAOMT3* proteins in multiple lines. Similar behavior is seen in the transgenics that individually knocked down *Ptr4CL3* (S4A Fig) and *Ptr4CL5* (S4B Fig), with significant decreases observed in the *PtrHCT1*, *PtrHCT6*, *PtrCCoAOMT3*, and *PtrCAD1* proteins. The *PtrHCT1*, *PtrHCT6*, *Ptr4CL3*, and *PtrCAD1* proteins are also significantly decreased in the *PtrCCoAOMT3* transgenics (Fig 2E). There are multiple transgenics where one line showed significant changes in all or almost all of the monolignol transcripts and proteins, but not in the other lines for the same transgenic such as i35-7 (Fig 2C), i15-3 (Fig 2D), i19-7 (S3F Fig), and a13-6 (S4B Fig). This behavior could be due to a nonlinear response to a change in the abundance of one or more of the monolignol transcripts and proteins.

Some of the observed indirect effects occur within gene families, such as in the *PtrPAL* knockdowns (S1A–S1D Fig), the *PtrCCoAOMT1* knockdowns (S3C Fig), the *PtrCAld5H1* and *PtrCAld5H2* single knockdowns (S3D and S3E Fig), and in the *Ptr4CL3* and *Ptr4CL5* single knockdowns (S4A and S4B Fig). These indirect effects within gene families could be due to sequence relationships with the targeted gene instead of regulatory mechanisms.

Capturing the effect of these indirect regulatory influences is necessary to effectively estimate the resulting protein levels that are responsible for driving monolignol biosynthesis. Further, it is necessary to capture the indirect effects that affect the transcripts and the indirect effects on the proteins separately.

### Computational model

**Fig 3:**
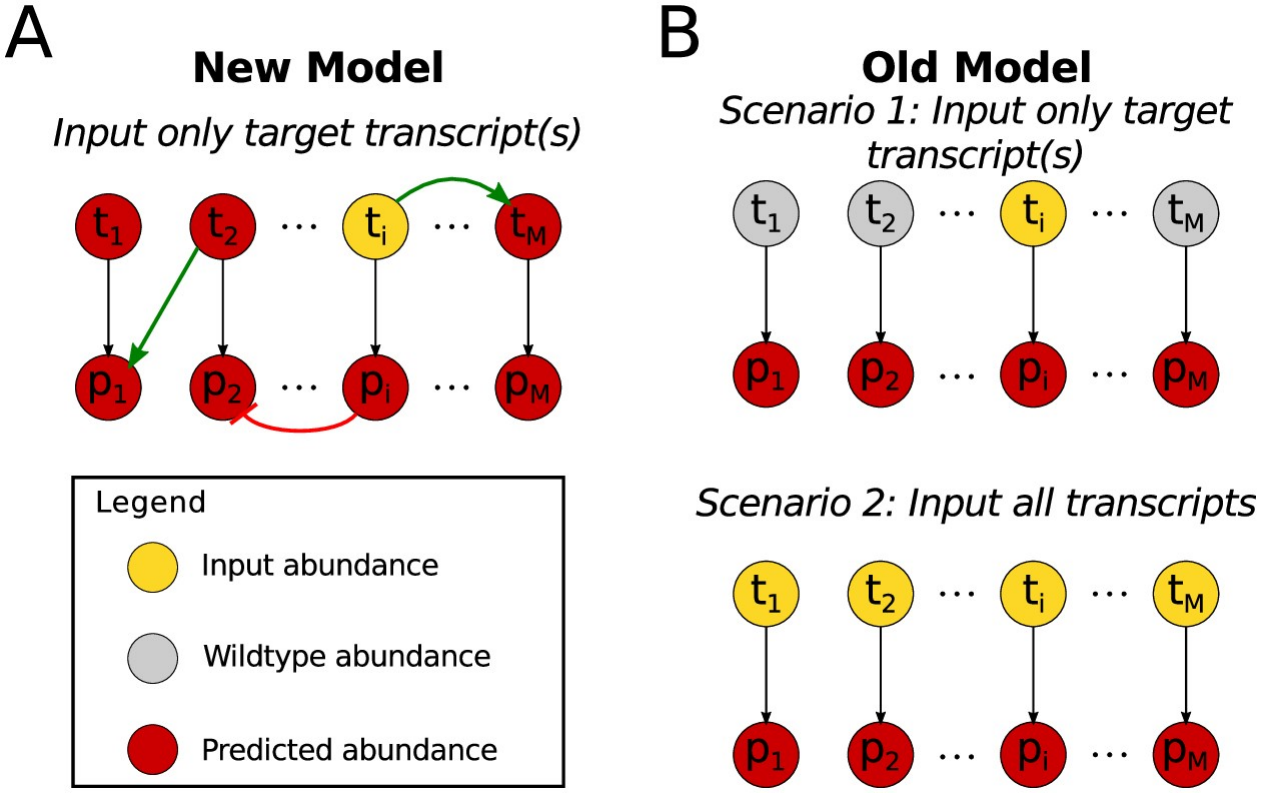
Diagram of transcript-protein models. (A) Diagram describing our model which includes positive (green arrows) and negative (red arrows) influences among the monolignol transcripts and proteins defined by **B** (Eq (3)). Using only targeted input abundances (yellow), the other untargeted monolignol transcripts and proteins are predicted (red) (B) In the old model only the one-to-one relationships from a monolignol transcript to its protein were included. In scenario 1, only the targeted monolignol transcripts were used as input abundances (yellow), the untargeted transcripts remained at wildtype levels (gray) and the protein abundances were predicted (red). In scenario 2, all of the monolignol transcript abundances were used as input (yellow) to predict (red) the monolignol protein abundances.

We developed a computational model that describes the observed cross-talk or interactions among the monolignol genes by representing each monolignol transcript and protein as a linear combination of the other monolignol transcripts and proteins. This formulation allows us to describe the indirect cross-influences as transcript to transcript and protein to transcript influences to represent influences impacting transcription, and transcript to protein and protein to protein influences to represent the indirect influences affecting the protein abundances. We estimated the weights of the connections that make up these linear combinations using a sparse maximum likelihood algorithm and the mean abundances from the experimental lines (see Methods and S1 Text). Using this model, we simulated the response of the untargeted monolignol gene transcripts and proteins based on the desired transcript abundance of a targeted monolignol gene or gene family (Fig 3A). We compare our model with the model from Wang et al. [4] which assumed that all of the protein abundances were proportional to their transcript levels (Fig 3B). We compare our model to two specific scenarios of this old model: scenario 1, where the desired targeted transcript levels are specified and the untargeted transcripts remain at wildtype levels, and scenario 2 where the full transcript profile is specified. We estimate the untargeted monolignol transcript and protein abundances using our model and both scenarios of the old model for single gene and gene family knockdowns corresponding to the transgenic experiments [4]. When exploring novel combinatorial knockdowns, however, where complete transcript profiles are unknown, scenario 2 cannot be simulated. We refer to the transcript of a gene as tGENE and the protein of a gene as pGENE in the following sections.

**Fig 4:**
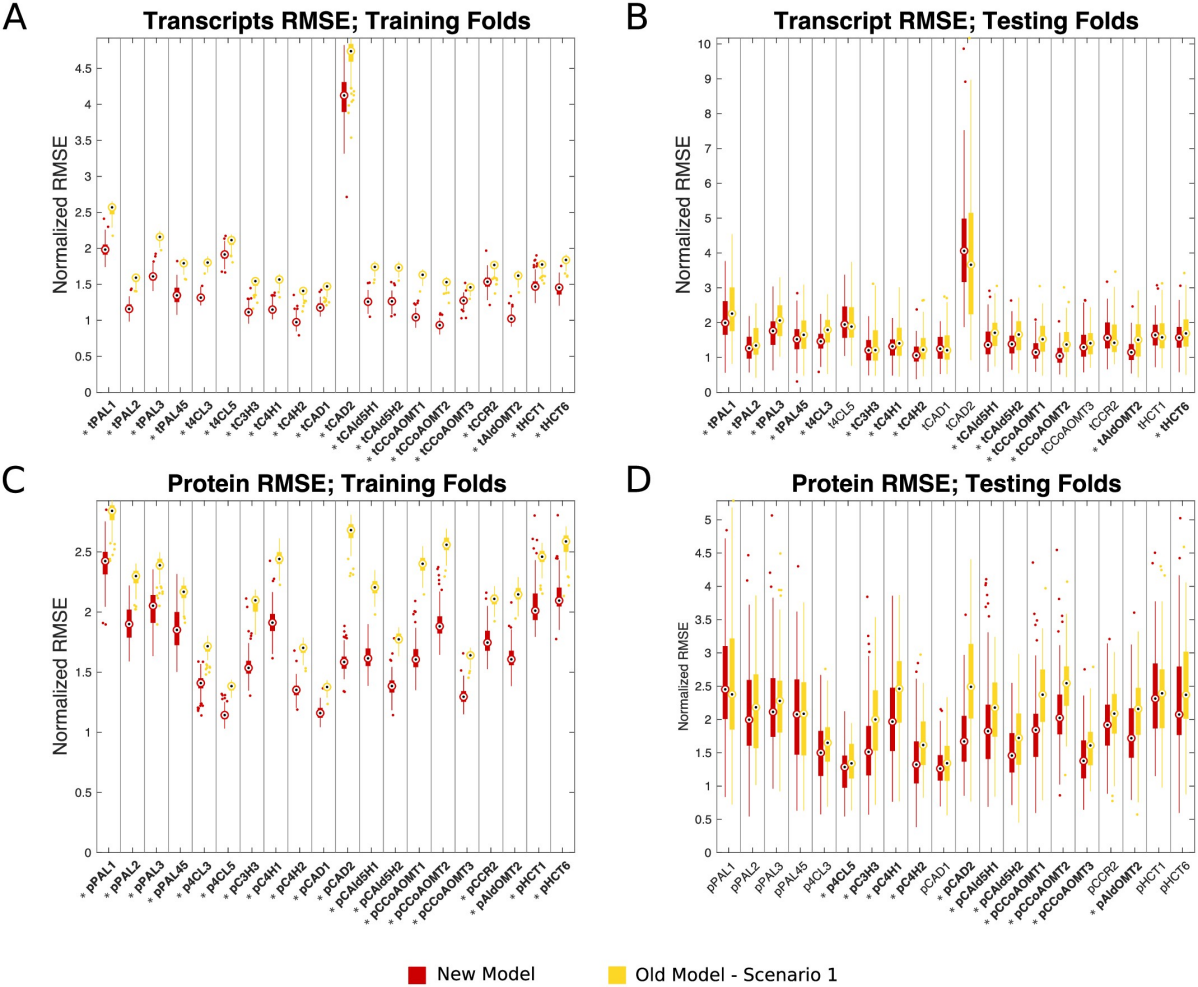
Boxplots of the RMSEs from the 10x10-fold cross-validation. Central marks indicate the medians and the bottom and top edges of each box indicate the 25th and 75th percentiles respectively. For these plots we normalized the RMSE for each monolignol transcript and protein by their corresponding standard deviations estimated from the wildtype experiments. This normalization allows each of the monolignol transcripts and proteins to be viewed on similar scales. Since the RMSEs from both models are scaled the same, this does not alter the interpretation of the results. Red boxes are from our new model, and the yellow boxes are from the old model. (A) Transcripts: training folds. (B) Transcripts: testing folds. (C) Proteins: training folds. (D) Proteins: testing folds.

We performed a 10x10-fold cross-validation resulting in 100 training and testing folds. The proposed model and the old model were trained on each of the 100 training folds. For each of the trained models, the knockdown experiments in the training fold and corresponding testing fold were emulated following the model estimation procedure (see Methods) for our model, and following scenario 1 for the old model. In each of these emulated experiments, the trained models estimated the untargeted monolignol gene transcripts and proteins. Fig 4 shows boxplots of the resulting root mean square errors (RMSE) of the estimated abundances across the 100 training (Fig 4A and 4C) and 100 testing folds (Fig 4B and 4D) for both our proposed model and the old model (Fig 3A and 3B—scenario 1). We performed a t-test to compare the distributions of the RMSEs from the new model and the old model for each monolignol transcript and protein. The x-axis labels with an asterisk had a significant difference (*p* <0.05) in the means of the distributions from the new model (red) and scenario 1 of the old model (yellow). The RMSEs for each transcript and protein were consistent between the testing sets and training sets (Fig 4), and 14 out of 20 of the transcripts and 11 out of 20 of the proteins in the testing sets were shown to have a significant difference in their predicted RMSEs (Fig 4B and 4D). In each of the significant cases, the distributions from the new model have a lower mean predicted RMSE. These cross-validation results show that our model performs as well or better than the scenario 1 of the old model.

**Fig 5:**
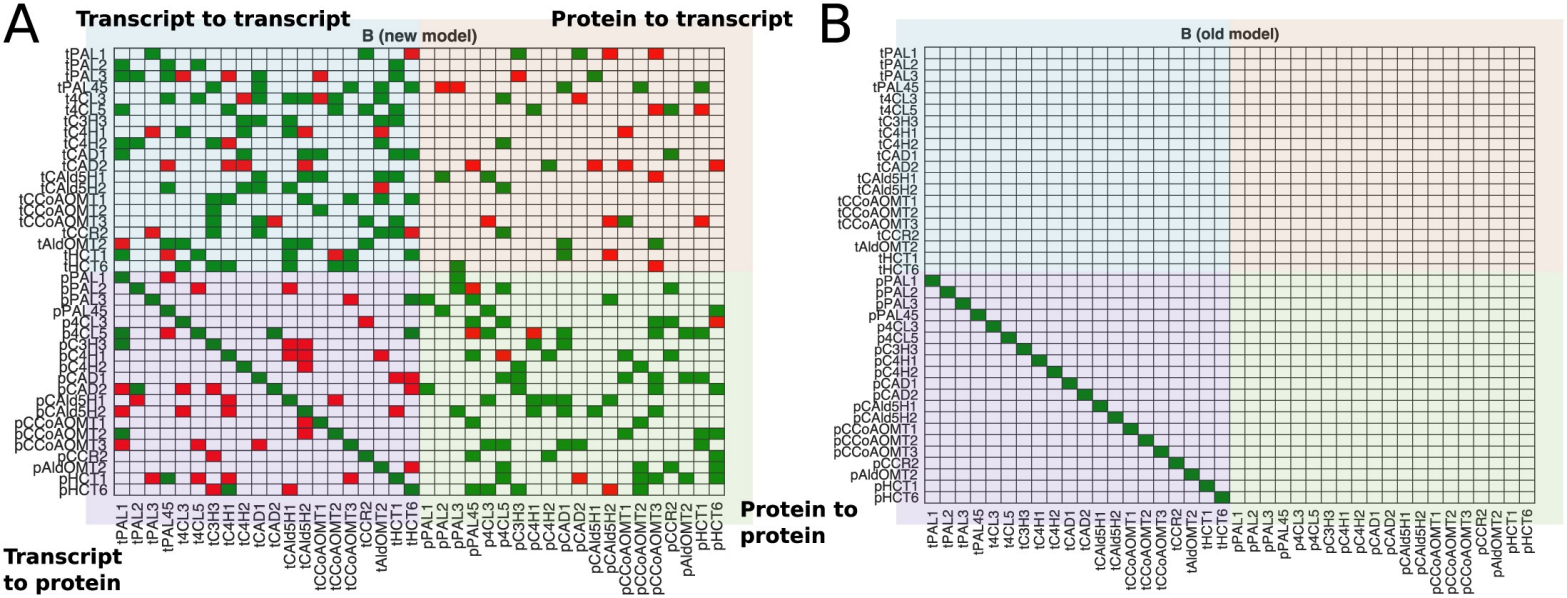
Heatmaps of the relationships in the transcript-protein models. (A) Heatmap of the edge matrix **B** (Eq 3) solved using a sparse maximum likelihood estimator. Green for positive influence, red for negative influence. Edges are from columns to rows (e.g., the first row shows edges tPAL3 → tPAL1, tCCR2 → tPAL1, tHCT6 → tPAL1, pC3H3 → tPAL1, pCAD2 → tPAL1, pCAld5H2 → tPAL1, and pCCoAOMT3 → tPAL1). There were 295 edges detected out of a possible 1540 (19.16% sparse). (B) The corresponding heatmap for the relationships considered in the old model (t_*i*_ → p_*i*_).

Fig 5A shows a heatmap of the relationships identified in our model (**B** in Eq 3) when trained on the means from all of the experimental lines. Green represents a positive influence, and red represents a negative influence. Each column represents the transcript or protein that is the source of an influence, and the row represents the transcript or protein that is being influenced. The top left quadrant contains the transcript to transcript influences, the top right quadrant contains the protein to transcript influences, the bottom left quadrant contains the transcript to protein influences, and the bottom right quadrant contains the protein to protein influences. There were 295 relationships detected out of a possible 1540 (19.16% sparse). The full set of relationships and their weights for our model can be found in S1 Table. For comparison, Fig 5B shows the equivalent representation of the old model, which just contains the *t*_*i*_ → *p*_*i*_ relationships. As expected, a positive influence was detected for each transcript to its associated protein (*t*_*i*_ → *p*_*i*_). The transcript to transcript and protein to protein influences make up the majority of the remaining influences estimated. There are not many protein to transcript influences detected, suggesting that protein abundances that are altered due to post-transcriptional or post-translational mechanisms may not result in changes at the transcriptional level that you would see with a targeted knockdown of that gene. Such as when the abundance of pCAD1 is decreased in the *PtrCCoAOMT3* (Fig 2E), *Ptr4CL3* (S4A Fig), or *Ptr4CL5* (S4B Fig) knockdowns, but the changes in transcript abundance that occur when *PtrCAD1* is knocked down (Fig 2C, S2C Fig) are not observed.

Through the cross-validation analysis, we showed that our new model is able to improve on the average estimation over all of the transgenic knockdowns in 14 of the transcripts and 11 of the proteins. However, we are more interested in looking at the specific transgenics where we are able to improve our prediction of the untargeted monolignol transcripts and proteins. To further evaluate how well our model captures these cross-influences affecting the monolignol transcript and protein abundances, we used our model and scenarios 1 and 2 of the old model to emulate the five transgenic experiments from our differential abundance analysis. For each of the five targeted experiments, we further described the results from the models for a subset of the untargeted monolignol genes that had a significant change in the abundance of their transcripts, proteins, or both in the differential abundance analysis.

#### *PtrC3H3*, *PtrC4H1*, and *PtrC4H2* knockdowns

**Fig 6:**
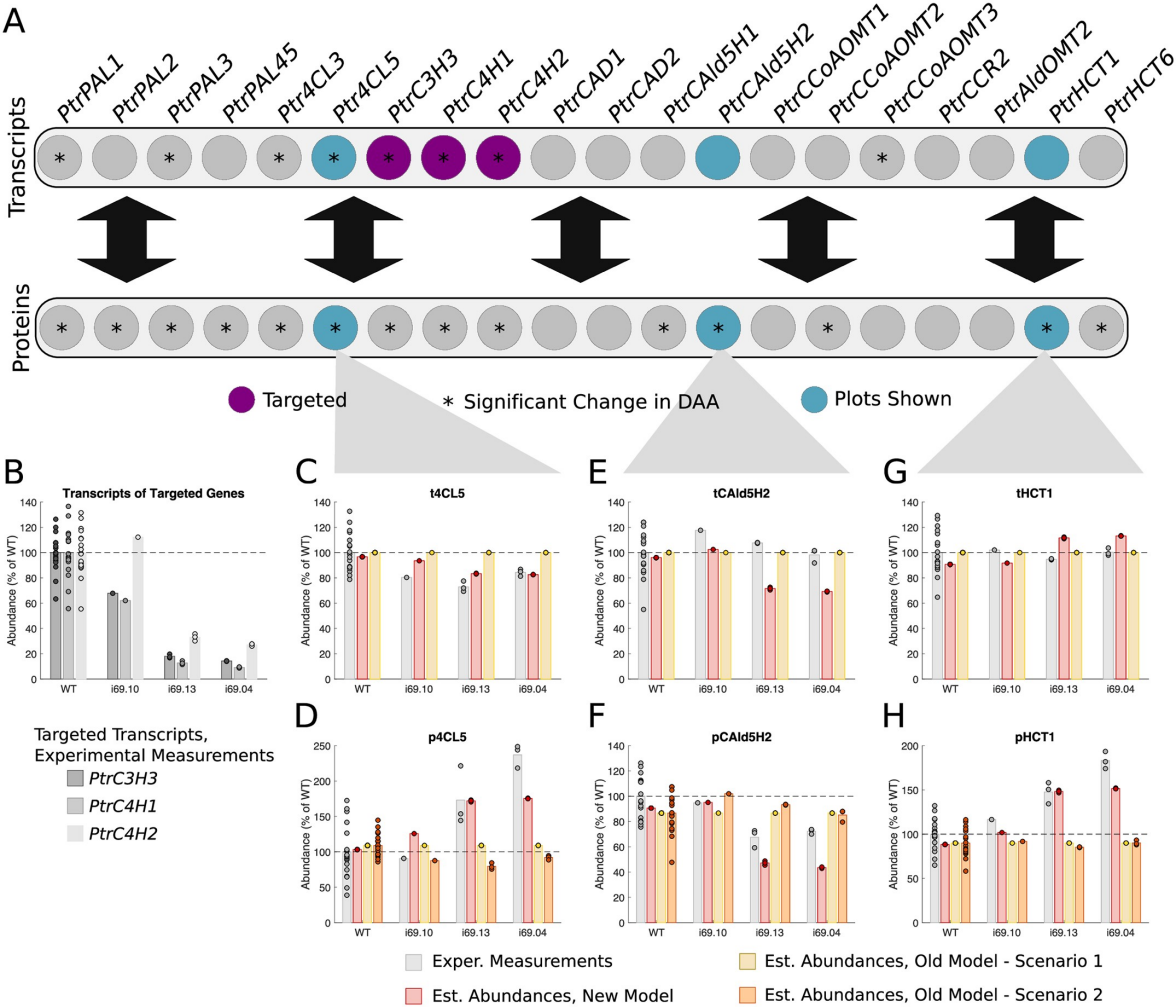
Experimental and estimated abundances of untargeted monolignol gene transcripts and proteins under *PtrC3H3*, *PtrC4H1*, and *PtrC4H2* knockdowns. (A) Diagram showing targeted monolignol gene transcripts (purple), the transcripts and proteins that were found to have a significant change in abundance in at least one of the experimental lines (*). (B) Level of knockdown of the targeted gene transcripts across the experimental lines. Experimental and estimated untargeted monolignol gene transcript and protein abundances for (C) t4CL5, (D) p4CL5, (E) tCAld5H2, (F) pCAld5H2, (G) tHCT1, and (H) pHCT1.

Three experimental lines were analyzed where *PtrC3H3*, *PtrC4H1*, and *PtrC4H2* were knocked down (Fig 6). From the differential abundance analysis, 5 transcripts and 11 proteins of the untargeted genes had a significant change in abundance in at least one of the experimental lines, which are signified by asterisks (Figs 2A and 6A). We include significant changes that occur in at least one of the lines since each line represents a different amount of knockdown of the targeted genes. We selected *Ptr4CL5*, *PtrCAld5H2*, and *PtrHCT1* transcripts and proteins to compare the simulated results from our model with scenarios 1 and 2 of the old model. The three targeted transcript abundances ranged from ∼110% to ∼10% of wildtype levels over the three experimental lines (Fig 6B). These tC3H3, tC4H1, and tC4H2 abundances were used to emulate these knockdown experiments in our model and scenario 1 of the old model. For scenario 2 of the old model, measurements from all of the monolignol transcripts were used.

Our model correctly estimated an increase in the *Ptr4CL5* and *PtrHCT1* proteins to ∼175% and ∼180% of wildtype levels respectively (Fig 6D and 6H), and a decrease in the *PtrCAld5H2* protein to ∼45% of wildtype levels (Fig 6F). In contrast, neither scenario of the old model captured these changes in the three proteins. Our new model also correctly estimated a decrease in t4CL5 (Fig 6C) and estimated tHCT1 to remain around wildtype levels (Fig 6G). It did, however, predict a slight decrease in tCAld5H2 abundance, which was not observed experimentally (Fig 6E). Note that scenario 1 of the old model assumes all untargeted transcripts remain at wildtype, while scenario 2 uses all of the experimental transcript abundances as inputs when predicting the protein abundances. As such, these models are not able to estimate transcript abundances.

#### *PtrCAld5H1* and *PtrCAld5H2* knockdowns

**Fig 7:**
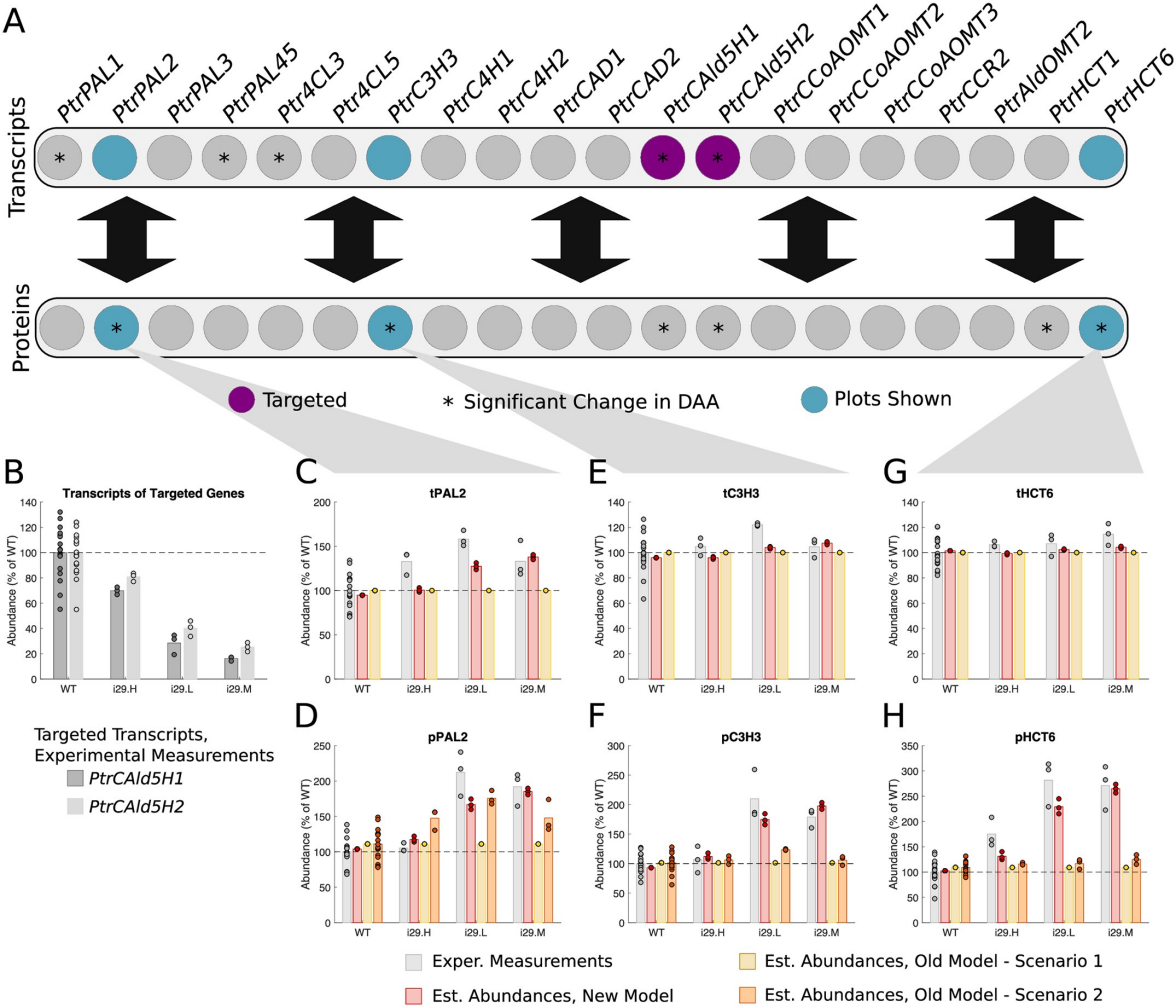
Experimental and estimated abundances of untargeted monolignol gene transcripts and proteins under *PtrCAld5H1* and *PtrCAld5H2* knockdowns. (A) Diagram showing targeted monolignol gene transcripts (purple), the transcripts and proteins that were found to have a significant change in abundance in at least one of the experimental lines (*). (B) Level of knockdown of the targeted gene transcripts across the experimental lines. Experimental and estimated untargeted monolignol gene transcript and protein abundances for (C) tPAL2, (D) pPAL2, (E) tC3H3, (F) pC3H3, (G) tHCT6, and (H) pHCT6.

Three experimental lines were analyzed where *PtrCAld5H1* and *PtrCAld5H2* were knocked down to the values seen in the experimental constructs (Fig 7). From the differential abundance analysis, there were 3 transcripts and 4 proteins of untargeted genes that showed significant changes in abundance in at least one of the experimental lines (Figs 2B and 7A). From these, we selected the *PtrPAL2*, *PtrC3H3*, and *PtrHCT6* transcripts and proteins to compare the simulated results from our model with scenarios 1 and 2 of the old model. Fig 7B shows the levels of knockdown, ranging from ∼80% to ∼20% of wildtype levels, for each of the three lines for the *PtrCAld5H1* and *PtrCAld5H2* transcripts. These tCAld5H1 and tCAld5H2 abundances were used to emulate these knockdown experiments in our model and scenario 1 of the old model. For scenario 2 of the old model, measurements from all of the monolignol transcripts were used.

Our model captured the increase from wildtype in all three proteins, pPAL2, pC3H3, and pHCT6, up to ∼185%, ∼200%, and ∼265% of wildtype levels, respectively (Fig 7D, 7F and 7H). Neither scenario of the old model captured the increase in pC3H3 or pHCT6 (Fig 7F and 7H). Scenario 2 of the old model estimated the increase in pPAL2 similar to the estimates from our model (Fig 7D). Additionally, the estimates from our model were consistent with the experimental tC3H3 and tHCT6, which were measured to remain around wildtype levels (Fig 7E and 7G). Our model also correctly estimated an increase in tPAL2 abudance (Fig 7C).

#### *PtrCAD1* and *PtrCAD2* knockdowns

**Fig 8:**
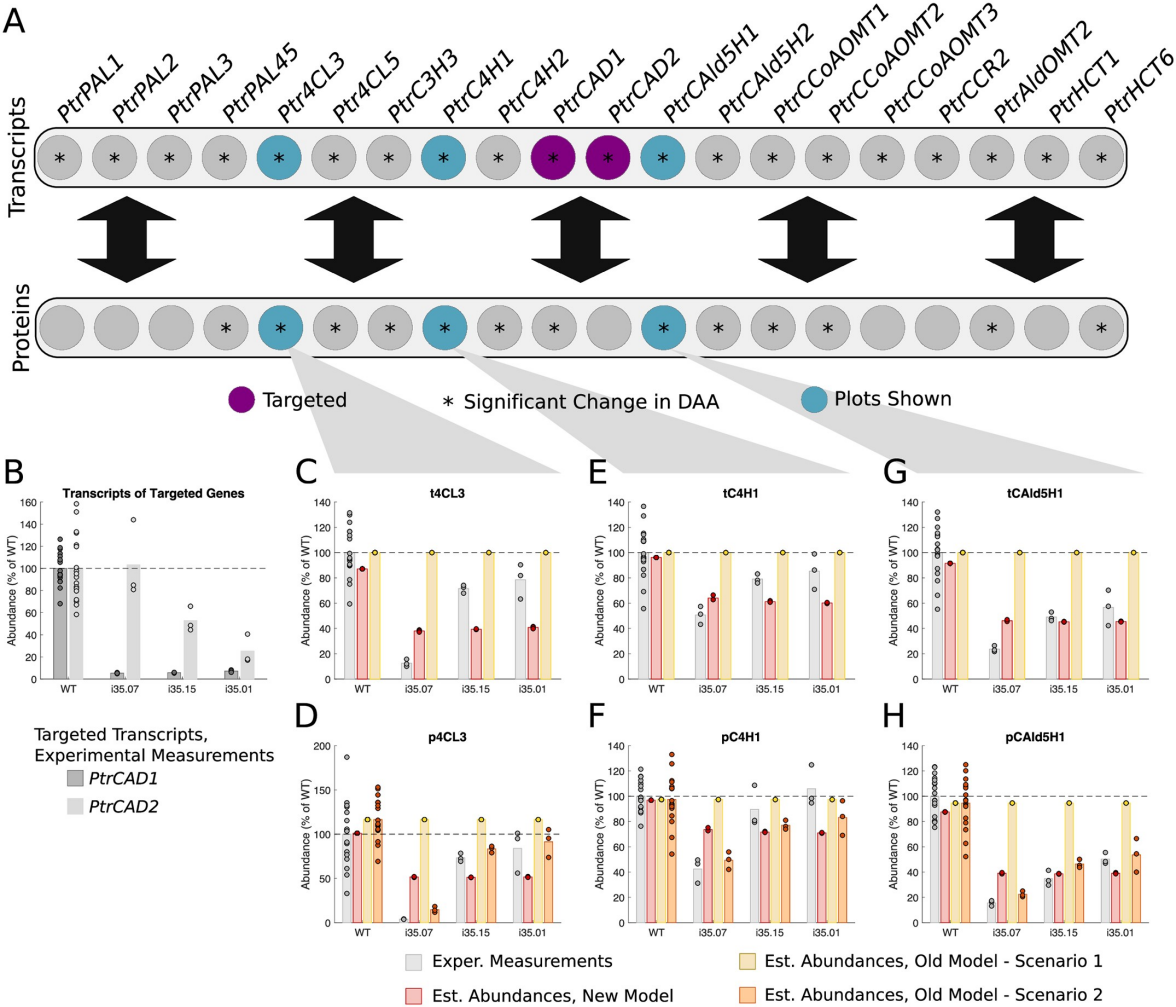
Experimental and estimated abundances of untargeted monolignol gene transcripts and proteins under *PtrCAD1* and *PtrCAD2* knockdowns. (A) Diagram showing targeted monolignol gene transcripts (purple), the transcripts and proteins that were found to have a significant change in abundance in at least one of the experimental lines (*). (B) Level of knockdown of the targeted gene transcripts across the experimental lines. Experimental and estimated untargeted monolignol gene transcript and protein abundances for (C) t4CL3, (D) p4CL3, (E) tC4H1, (F) pC4H1, (G) tCAld5H1, and (H) pCAld5H1.

Three experimental lines were analyzed where *PtrCAD1* and *PtrCAD2* were knocked down (Fig 8). From the differential abundance analysis, there were 18 transcripts and 12 proteins of untargeted genes that showed significant changes in abundance in at least one of the experimental lines (Figs 2C and 8A). We selected the *Ptr4CL3*, *PtrC4H1*, and *PtrCAld5H1* transcripts and proteins to compare the simulated results from our model with scenarios 1 and 2 of the old model. Fig 8B shows the amount that tCAD1 and tCAD2 were knocked down in the three experimental lines. For all three of these lines, tCAD1 was knocked down to ∼5% of wildtype levels while tCAD2 ranged from no change from wildtype to ∼25% of wildtype. These tCAD1 and tCAD2 abundances were used to emulate these knockdown experiments in our model and scenario 1 of the old model. For scenario 2 of the old model, measurements from all of the monolignol transcripts were used.

In this case, scenario 2 of the old model did the best at estimating all three of the proteins (Fig 8D, 8F and 8H) because the decrease was captured in the transcript abundances. However, our model still captured the decrease from wildtype in both the transcripts and proteins (Fig 8C–8H) despite only using the *PtrCAD1* and *PtrCAD2* transcript abundances as inputs to the model. The estimates from our model for the transcripts and proteins are very similar across the three experimental lines. This is due to the sparse maximum likelihood algorithm identifying *PtrCAD1*, which was knocked down similarly for all three lines, as a stronger influence on the other transcripts and proteins than *PtrCAD2*.

#### *Ptr4CL3* and *Ptr4CL5* knockdowns

**Fig 9:**
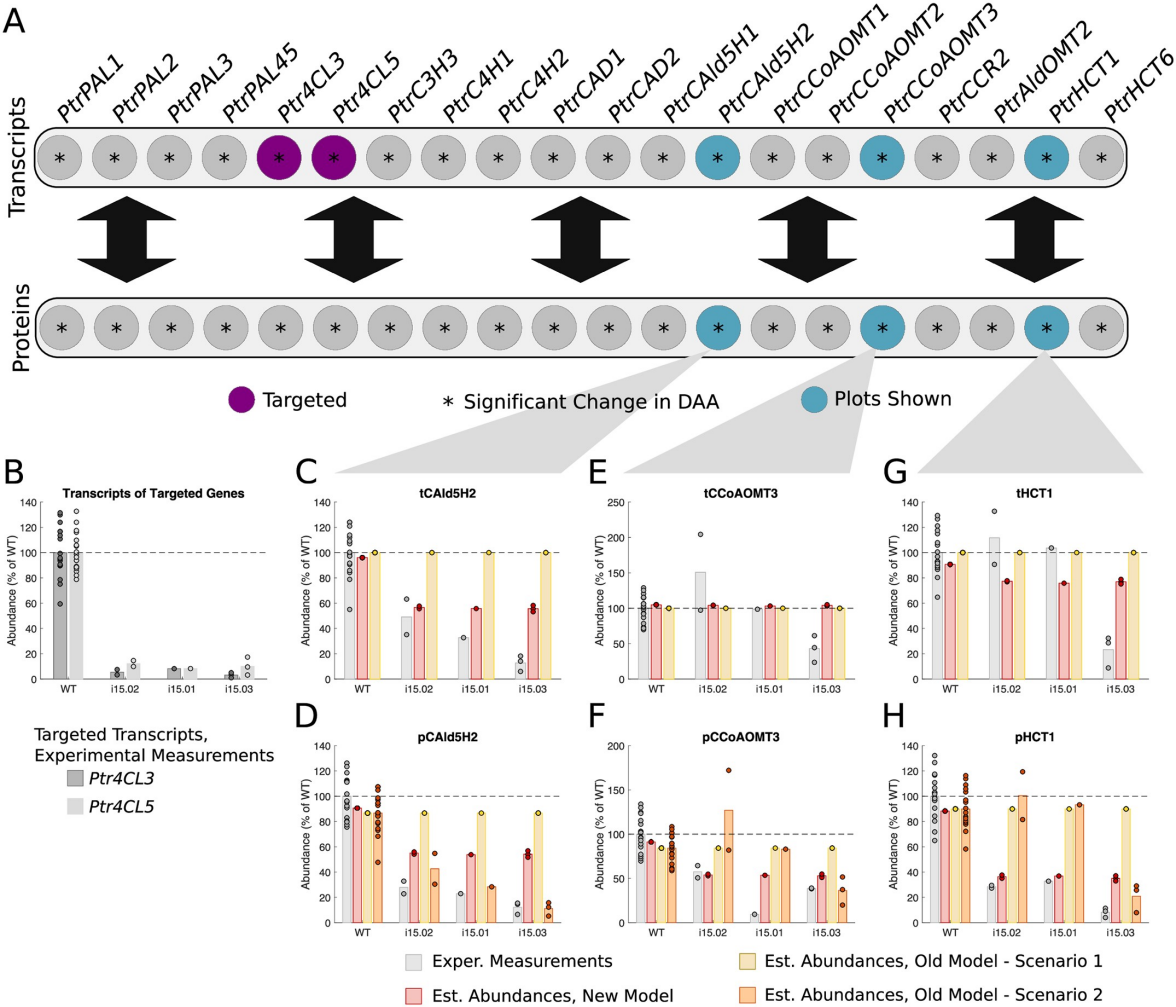
Experimental and estimated abundances of untargeted monolignol gene transcripts and proteins under *Ptr4CL3* and *Ptr4CL5* knockdowns. (A) Diagram showing targeted monolignol gene transcripts (purple), the transcripts and proteins that were found to have a significant change in abundance in at least one of the experimental lines (*). (B) Level of knockdown of the targeted gene transcripts across the experimental lines. Experimental and estimated untargeted monolignol gene transcript and protein abundances for (C) tCAld5H2, (D) pCAld5H2, (E) tCCoAOMT3, (F) pCCoAOMT3, (G) tHCT1, and (H) pHCT1.

Three experimental lines were analyzed where *Ptr4CL3* and *Ptr4CL5* were knocked down (Fig 9). The differential abundance analysis identified 18 transcripts and 18 proteins of untargeted monolignol genes that showed significant changes in abundance in at least one of the experimental lines (Figs 2D and 9A). We selected the *PtrCAld5H2*, *PtrCCoAOMT3*, and *PtrHCT1* transcripts and proteins to compare the simulated results from our model with scenarios 1 and 2 of the old model. Fig 9B shows the different levels that t4CL3 and t4CL5 were knocked down for the three experimental lines. For all three of the lines, the transcripts were knocked down to around the same levels, ∼5%-10% of wildtype levels. These t4CL3 and t4CL5 abundances were used to emulate these knockdown experiments in our model and scenario 1 of the old model. For scenario 2 of the old model, measurements from all of the monolignol transcripts were used.

For all three of the proteins, our model correctly estimated a decrease from their wildtype abundances. Our model predicted a decrease down to ∼55% of wildtype for pCAld5H2 and pCCoAOMT3 (Fig 9D and 9F) and to ∼40% of wildtype levels for pHCT1 (Fig 9H). Scenario 2 of the old model did a better job of capturing the decrease in pCAld5H2 estimating a decrease ranging from ∼40% to ∼10% wildtype levels (Fig 9D), but only estimated a decrease in the third line for both pCCoAOMT3 and pHCT1 (Fig 9F and 9H). Our model also captured the decrease in tCAld5H2 (Fig 9C), and its estimates for tCCoAOMT3 and tHCT1 (Fig 9E and 9G) are reasonable considering the range of the measured abundances across the three lines.

#### *PtrCCoAOMT3* knockdowns

**Fig 10:**
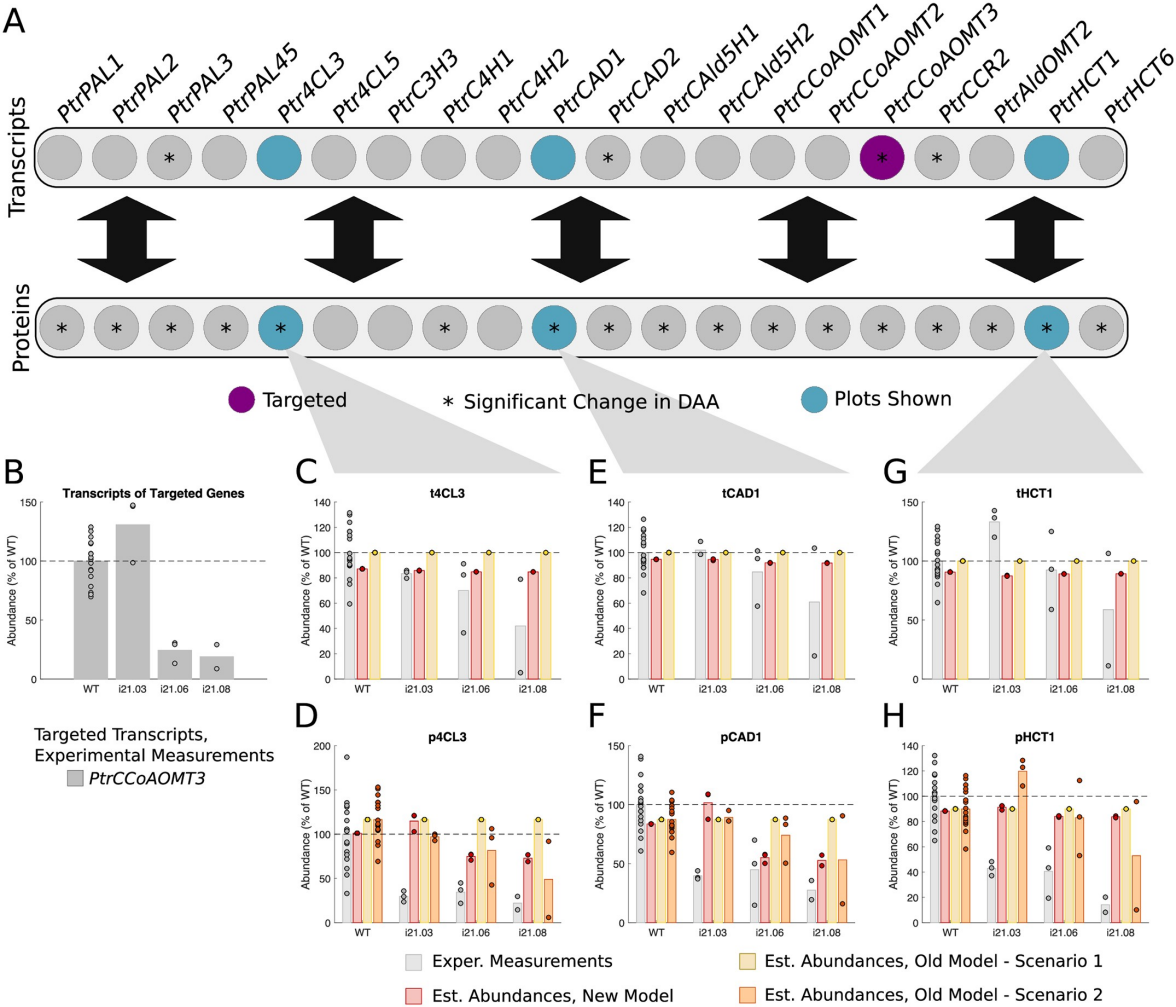
Experimental and estimated abundances of untargeted monolignol gene transcripts and proteins under *PtrCCoAOMT3* knockdowns. (A) Diagram showing targeted monolignol gene transcripts (purple), the transcripts and proteins that were found to have a significant change in abundance in at least one of the experimental lines (*). (B) Level of knockdown of the targeted gene transcripts across the experimental lines. Experimental and estimated untargeted monolignol gene transcript and protein abundances for (C) t4CL3, (D) p4CL3, (E) tCAD1, (F) pCAD1, (G) tHCT1, and (H) pHCT1.

Three experimental lines were analyzed where *PtrCCoAOMT3* was knocked down (Fig 10). The differential abundance analysis identified 3 transcripts and 16 proteins of untargeted monolignol genes that had significant changes in abundance in at least one of the experimental lines (Figs 2E and 10A). We selected the *Ptr4CL3*, *PtrCAD1*, and *PtrHCT1* transcripts and proteins to compare the simulated results from our model with scenarios 1 and 2 of the old model. Fig 10B shows the range that tCCoAOMT3 was knocked down over the 3 experimental lines. In the first line, i21-03, tCCoAOMT3 was not knocked down from wildtype. In the other two lines it was knocked down to ∼20% of wildtype levels. These tCCoAOMT3 abundances were used to emulate these knockdown experiments in our model and scenario 1 of the old model. For scenario 2 of the old model, measurements from all of the monolignol transcripts were used.

Neither our model, nor the old model, did a good job at estimating the experimentally observed changes for the *Ptr4CL3* and *PtrHCT1* transcripts and proteins (Fig 10C, 10D, 10G and 10H). However, our model did do a better job of capture the decrease in pCAD1, estimating a decrease to ∼55% wildtype levels in two of the three lines (Fig 10F).

### Analysis of network topology

We further examined the specific connections identified in **B** from Eq 3, by identifying the edges that contribute most to changing a transcript or protein abundance from its wildtype abundance. For each transcript and protein, we identified the transgenic constructs where (1) our model correctly estimated the results within a certain tolerance, and (2) the transcript or protein was differentially expressed in at least one of the experimental lines (Fig 2, S1–S4 Figs). We then computed the difference between the contribution of each relationship in a wildtype simulation and in the transgenic simulation. The edges that did not contribute at least ±50% of the net change were removed. After this filtering, 159 of the original 295 edges remained to make up a network of the influences that contributed most to a transcript or proteins change from wildtype abundance.

**Fig 11:**
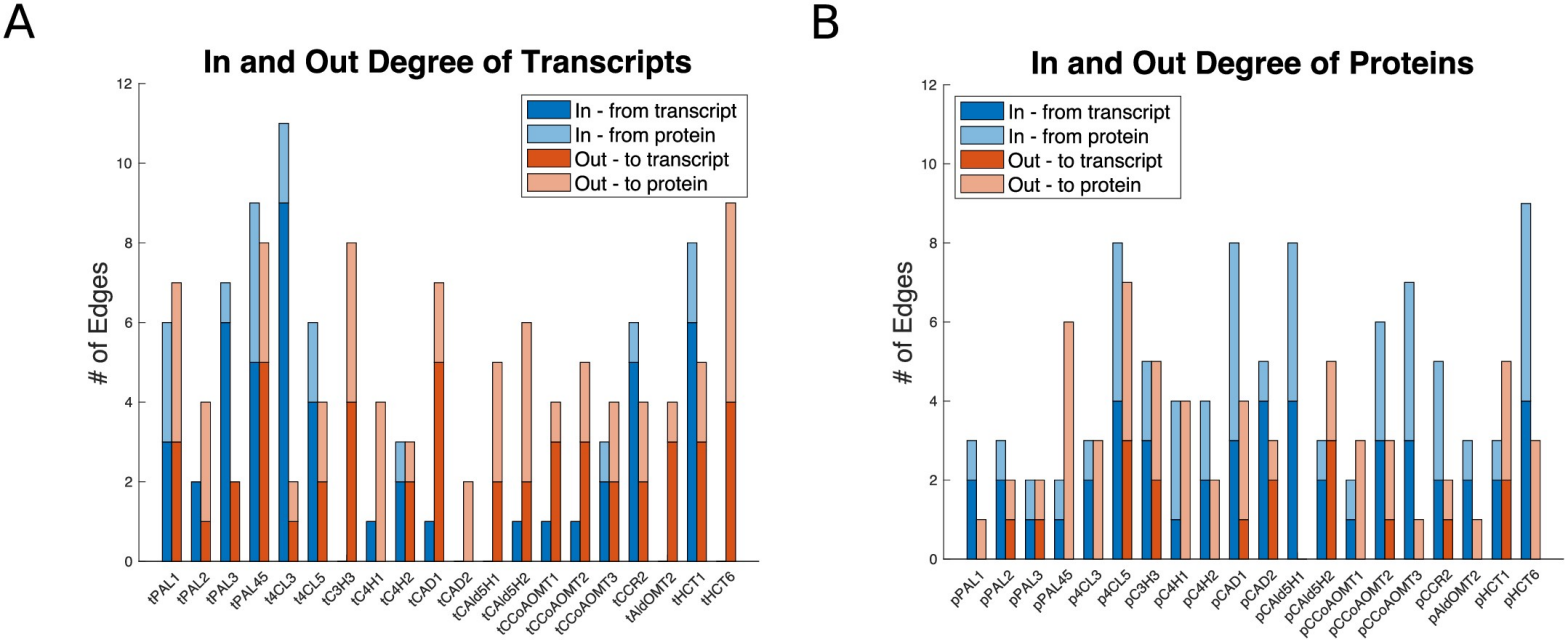
In-degrees and Out-degrees for the monolignol transcripts and proteins. (A) Transcripts. (B) Proteins. Dark blue—Edges originating from a transcript; Light blue—Edges originating from a protein; Dark Orange: Edges going to a transcript; Light orange—Edges going to a protein.

In this network, the transcripts were generally the source of more edges than the target, with several transcripts only having outgoing edges (Fig 11A, tC3H3, tCAD2, tCAld5H1, tAldOMT2, and tHCT6). This suggests that these transcripts are less likely to be altered in transgenics where they were not the target. The median out-degree of the transcripts was 4 edges and the median in-degree was 1.5 edges. The *PtrPAL*, *Ptr4CL*, *PtrCCR2*, and *PtrHCT1* transcripts had the most incoming edges of all the transcripts, with most of their edges coming from other transcripts (Fig 11A). The outgoing edges for the transcripts were split almost evenly to edges going to transcripts and edges going to proteins, indicating that changing transcript abundances results in altering both other transcript abundances and some protein abundances separately of their associated transcript.

The proteins (Fig 11B) were generally the target of more edges than the source with a median in-degree of 4 edges and a median out-degree of 3 edges. The proteins were influenced about equally by transcripts (∼52% of incoming edges) and other proteins (∼48% of incoming edges), but had more edges influencing other proteins (∼73% of outgoing edges) than transcripts (∼27% of outgoing edges).

The *PtrPAL* family had more incoming edges impacting their transcript abundances than their protein abundances, suggesting that they were mostly influenced at the transcriptional level. The *PtrC3H3*, *PtrC4H*, *PtrCAD*, *PtrCAld5H*, and *PtrCCoAOMT* families had more incoming edges impacting their protein abundances, suggesting that they may be more likely to be influenced post-transcriptionally or post-translationally. This follows our results from the differential abundance analysis where changes in the abundance of these proteins did not always track with changes in their transcript abundance (Fig 2). The *Ptr4CL* and *PtrHCT* families had incoming edges impacting both their transcripts and proteins, indicating that they are impacted at both transcription and after transcription.

**Fig 12:**
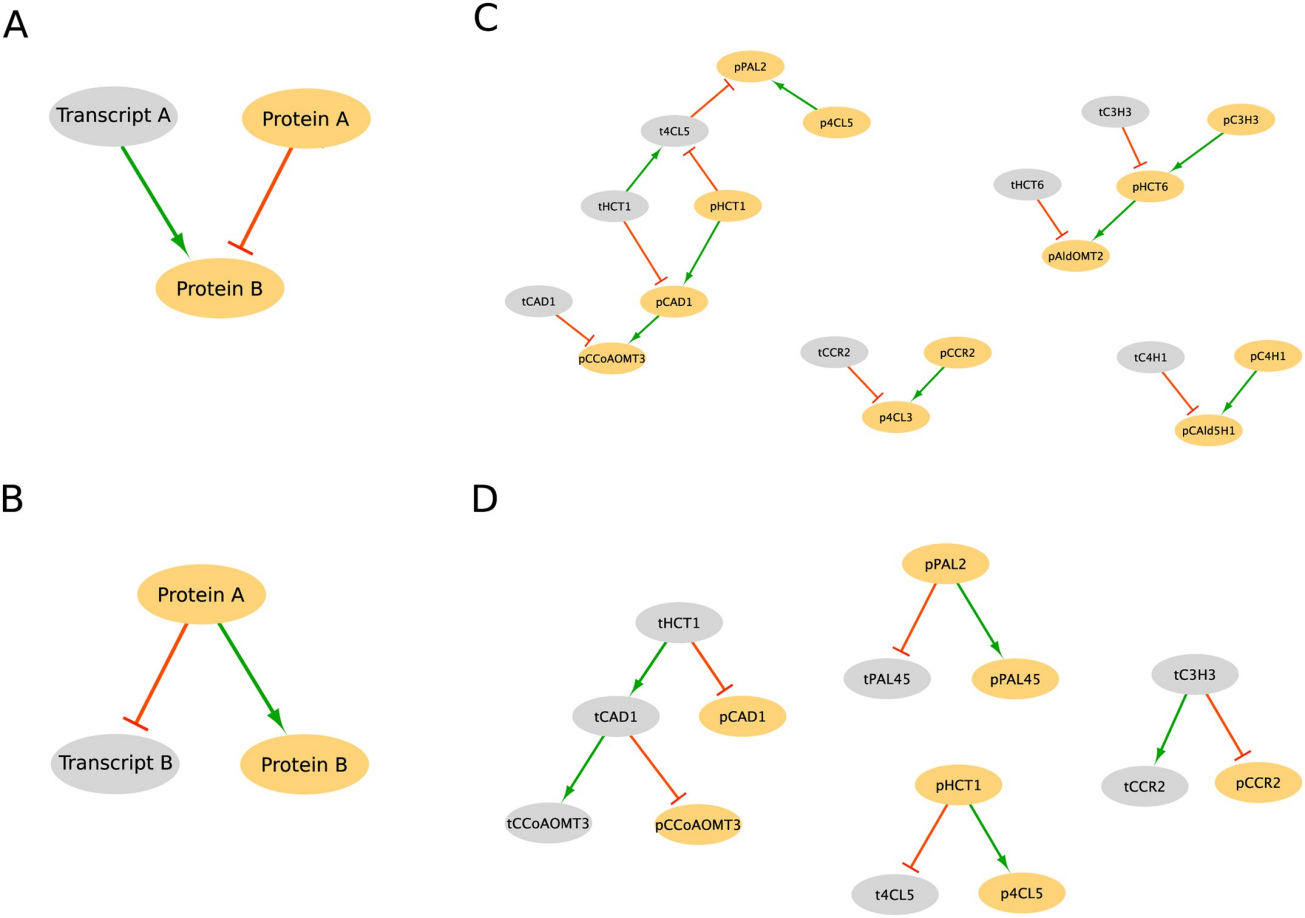
Network motifs indicating post-transcriptional or post-translational regulation. (A) Motif 1. (B) Motif 2. (C) Instances of Motif 1 in network. (D) Instances of Motif 2 in network.

We identified two network motifs that suggested possible post-transcriptional or post-translational regulatory influence. We define these motifs as Motif 1 and Motif 2. Motif 1 occurs when the transcript/protein pair of gene A affect the transcript (or protein) of gene B, and the influence of the transcript is opposite of the influence of the protein (Fig 12A). Motif 2 occurs when the transcript (or protein) from gene A influences both the transcript and protein of gene B, but the influence on transcript B by gene A is opposite the influence on protein B (Fig 12B). In Motif 1, when both the transcript and protein of gene A show a change in abundance, their influences combine to have little or no change on the abundance of the influenced transcript (or protein) B. Only when either the abundance of transcript A or the abundance of protein A are independently altered, such as from post-transcriptional or post-translational regulation, would there be a net change on the transcript (or protein) B. Similarly in Motif 2, a change in abundance of transcript (or protein) A results in a change in the abundance of transcript B but either no change, or a change in the opposite direction, in protein B. There were 8 instances of Motif 1 in our network (Fig 12C) and 5 instances of Motif 2 (Fig 12D).

Thirteen of the monolignol genes are represented at least once in these motifs, with the *PtrHCT*, *PtrCAD*, and *Ptr4CL* gene families being the most represented (Table 1). *PtrHCT1* is a source in all four of the motifs it is a member of, while *PtrCAD1*, *Ptr4CL5*, and *PtrHCT6* act as both sources and targets. This suggests that these three gene families are potential targets for further experimentation to elucidate the post-transcriptional and post-translational regulatory mechanisms at work.

**Table 1:** Appearance of monolignol genes in Motifs 1 and 2.

		*PtrCAD1*	*PtrHCT1*	*Ptr4CL5*	*PtrHCT6*	*PtrPAL2*	*PtrC3H3*	*PtrCCoAOMT3*	*PtrCCR2*	*Ptr4CL3*	*PtrC4H1*	*PtrCAld5H1*	*PtrAldOMT2*	*PtrPAL4/5*
**Motif 1**	**Source**	1	2	1	1	0	1	0	1	0	1	0	0	0
	**Target**	1	0	1	1	1	0	1	0	1	0	1	1	0
**Motif 2**	**Source**	1	2	0	0	1	1	0	0	0	0	0	0	0
	**Target**	1	0	1	0	0	0	1	1	0	0	0	0	1

## Discussion

We used the connections identified by the sparse maximum likelihood estimator to define our new transcript-protein model for monolignol biosynthesis. Using this model, we emulated the 225 wildtype and transgenic knockdown experiments using only the measured transcript abundances from the targeted monolignol genes as an input and estimating the abundances of the other, untargeted, transcripts and proteins. We compared these estimates to those found using the old model [4], which assumes the protein abundances are linearly proportional to the transcript abundance of the same monolignol gene. We performed a 10x10-fold cross-validation and compared the resulting RMSE distributions from the old model and our new model. The mean predicted RMSEs for 14 of the 20 transcripts and 11 of the 20 proteins were found to be statistically lower in our new model than the old model.

We simulated the transgenic experiments using our model and scenarios 1 and 2 of the old model, and compared the estimated transcript and protein abundances of selected untargeted genes of interest. As expected, scenario 2 of the old model, which uses the full transcript abundance profiles, did the best at estimating the proteins whose abundance levels tracked the abundance levels of its transcripts, such as *Ptr4CL3*, *PtrC4H1*, and *PtrCAld5H1* in the *PtrCAD1* and *PtrCAD2* knockdown experiments (Fig 8C–8H), and *PtrCAld5H2* in the *Ptr4CL3* and *Ptr4CL5* knockdowns (Fig 9C and 9D). However, using only the targeted *PtrCAD1* and *PtrCAD2* or *Ptr4CL3* and *Ptr4CL5* transcripts respectively, our model was still able to estimate the decreases in both the transcripts and proteins for all four of these genes. Additionally, our model was able to capture several changes in protein abundances that the old model was not, including *Ptr4CL5*, *PtrCAld5H2*, and *PtrHCT1* in the *PtrC3H3*, *PtrC4H1*, and *PtrC4H2* knockdowns; *PtrC3H3* and *PtrHCT6* in the *PtrCAld5H1* and *PtrCAld5H2* knockdowns; and *PtrCCoAOMT3* and *PtrHCT1* in the *Ptr4CL3* and *Ptr4CL5* knockdowns.

Neither model was able to estimate the changes in abundance of the *Ptr4CL3* and *PtrHCT1* proteins in the *PtrCCoAOMT3* transgenics. Our model includes relationships from pCCoAOMT3 to p4CL3 and pHCT1. Despite this, our model does not capture the size of the decrease in the abundances of these proteins. One explanation for why the extent of these regulatory influences are not captured in our simulations could be due to constraining the regulatory influences to additive linear relationships. Some of the shortcomings of an additive linear model include not allowing for nonlinear relationships and not being able to capture synergistic influence behaviors (i.e., when multiple components are needed to see an effect).

In our differential abundance analysis, we observed several instances of differential expression in untargeted transcripts and proteins. The *PtrCAD1* and *PtrCAD2* transgenics (Fig 2C) showed several changes in the untargeted monolignol transcript abundances and protein abundances across all three experimental lines. In our analysis of the network topology, we found that the *PtrCAD1* transcript is the source of several influences, especially to other transcripts (Fig 11A). These results suggest that knocking down *PtrCAD1* and *PtrCAD2* sets off a transcriptional regulatory response. Chen et al., recently constructed a hierarchical transcriptional regulatory network for wood formation in *P. trichocarpa*, identifying 7 transcription factors regulating 10 of the monolignol specific genes [12]. Most of these transcription factors were also found to be differentially expressed in the *PtrCAD1* and *PtrCAD2* transgenics (S5 Fig), further supporting that the cross-influences impacting the abundances of these transcripts are occurring through transcriptional regulation.

In addition to changes in transcript abundance, we also observed several cases where monolignol protein abundances were significantly altered when their transcripts were not (Fig 2, S1–S4 Figs), suggesting the presence of post-transcriptional or post-translational regulation. In our network topology analysis, ∼73% of the edges originating from a protein influenced another protein (Fig 11B), indicating that a change in the abundance of a protein is more likely to impact the abundance of another protein without influencing the abundance of the associated transcript. Further, 13 of the monolignol genes were either a source, target, or both in the two motifs that represent potential post-transcriptional or post-translational relationships (Fig 12). Compared to transcriptional regulation, less is known about the role of post-transcriptional and post-translational regulation in monolignol biosynthesis. Phosphorylation of the *PtrPAL* protein was proposed over two decades ago, though the role of this phosphorylation is unknown [36, 37]. Wang et al., [15] characterized the phosphorylation of the *PtrAldOMT2* protein in *P. trichocarpa*, finding it to impact its activity but not abundance. Loziuk et al., recently identified 12 monolignol proteins in *P. trichocarpa* that contain sequence motifs for glycosylation, a post-translational modification that can impact protein abundance levels [16]. Six of the genes they identified are represented at least once in the two network motifs from our model (*PtrHCT1*, *Ptr4CL3*, *PtrCCR2*, *PtrPAL4*, *PtrPAL5*, and *PtrC3H3*), and the other six genes have a family member represented in the motifs. The *PtrHCT*, *PtrCAD*, and *Ptr4CL* families were the most involved in the topological network motifs (Table 1), and were also represented in the genes containing a glycosylation motif [16]. We believe that these three monolignol gene families are good starting points for further experimentation to explore and identify the post-transcriptional or post-translational regulatory mechanisms responsible for the observed differential abundance behavior.

The monolignol proteins are the driving forces in the biosynthesis pathway. Being able to accurately understand and estimate how these proteins change under different combinations and degrees of targeted genetic modifications is important for the accuracy of predictive models. Regulatory influences that occur after transcription appear in the monolignol data of stem differentiated xylem tissue in *P. trichocarpa*, and we have developed a computational model that incorporates influences on both the monolignol transcripts and proteins. We have demonstrated specific examples where our model produces better estimates of experimental monolignol gene proteins than the old model when both models use only the targeted monolignol transcript abundances as input. In several cases our model, using only the targeted transcript abundances, produced better estimates than scenario 2 of the old model where all of the experimental transcript abundances were used. By incorporating these indirect regulatory influences, we believe our model has improved ability to explore the cascaded impact of genetic modifications on resulting lignin and wood characteristics. Additionally, we identified three gene families, *PtrHCT*, *PtrCAD*, and *Ptr4CL* that appear to be most involved in the post-transcriptional or post-translational influences, which could be further experimentally examined to elucidate the specific regulatory mechanisms responsible for the observed behavior.

The approach presented provides a phenological representation for predicting the transcript and protein abundances resulting from specific knockdowns of monolignol genes. This approach does not, however, capture nested causal relationships that are inherent to complex gene regulatory networks. Using this approach we were able to predict the impact of cross-influences between transcripts and proteins. Additionally, the results from the topological analysis provide insight into potential candidates for future experiments aimed at elucidating the specific regulatory mechanisms responsible for the observed cross-influences. Future work will evaluate how our model performs on independent data, incorporate the model into the multi-scale model in [4], and use the multi-scale model to explore the possible changes in lignin and wood characteristics under combinations of lignin gene modifications.

## Methods

### Monolignol transcript-protein model

The multi-scale lignin biosynthesis model presented in [4] spans multiple biological layers from the genome to observed lignin and wood physical and chemical traits. However, that model [4] makes the simplifying assumption that each monolignol gene’s protein abundance is dependent only on its transcript abundance. This does not reflect any changes that are observed in the abundance of the non-targeted genes. Here, we present a new model that incorporates the observed influences that estimate the production of untargeted monoligninol transcripts and proteins. The code associated with this model can be found at https://github.com/leighmatth/Monolignol-Cross-Regulation-Model.

Because we are interested in identifying regulatory influences at not only the transcriptional level, but also the translational level, we combined the two datasets, such that we are now looking at each of the 20 transcripts and 20 proteins as 40 total variables in our model.

#### Model development

The goal of the model development is to find the underlying influences on each monolignol gene product (its transcripts and proteins) when the expression of other monoligninol genes are modified. We describe each transcript and protein as a linear combination of the other transcripts and proteins as shown in Eq (1).

$$\begin{array}{c} y_{i}=\mu_{i}+B_{i1}y_{1}+\cdots +B_{ij}y_{j}+\cdots +B_{iM}y_{M}+\epsilon \;\forall j\neq i \end{array}$$ (1)

Where *y*_*i*_ is the abundance of the *i*^*th*^ gene product, and we have *M* total gene products ($\frac{M}{2}$ transcripts and $\frac{M}{2}$ proteins). *B*_*ij*_ is a constant term that reflects the influence of gene product *j* on gene product *i*, *μ*_*i*_ is a constant that represents the portion of *y*_*i*_ that is not described by the other lignin gene products, and *ϵ* is the error. The influences described by *B*_*ij*_ should be consistent across multiple experiments, so we can describe Eq (1) over a collection of experiments as shown in Eq (2).

$$\begin{array}{c} y_{i}^{T}=\mu_{i}1^{T}+B_{i1}y_{1}^{T}+\cdots +B_{ij}y_{j}^{T}+\cdots +B_{iM}y_{M}^{T}+\epsilon^{T}\;\forall j\neq i \end{array}$$ (2)

Where **y**_*i*_ ∈ ℜ^*N*^ is the abundances of *i*^*th*^ gene product over *N* experiments. We can combine this into one model for all the transcripts and proteins as shown in Eq (3).

$$\begin{array}{c} Y=\text{BY}+\mu 1^{T}+E,\;s.t.\;B_{ii}=0\;\forall i \end{array}$$ (3)

Where $Y=[\begin{array}{c} T\\ P \end{array}]\in {ℜ}^{M\times N}$ is a matrix composed of the abundances for the $\frac{M}{2}$ transcripts (**T**) and associated $\frac{M}{2}$ proteins (**P**) for each of the *N* experiments. **B** ∈ ℜ^*M*×*M*^ is the collection of influence terms *B*_*ij*_. Because each *y*_*i*_ is a function of the other gene products *y*_*j*_ ∀*j* ≠ *i*, the diagonal elements of **B**, *B*_*ii*_ = 0 ∀*i*. Additionally, we also enforce a constraint that a transcript cannot be influenced by its associated protein (*p*_*i*_ ↛ *t*_*i*_). ***μ*** ∈ ℜ^*M*^ is a vector containing a constant term for each gene product, and **1** ∈ ℜ^*N*^ is a vector of all ones. $E=[\begin{array}{cccc} \epsilon_{1} & \epsilon_{2} & \cdots & \epsilon_{N} \end{array}]$ represents the error where $\epsilon_{j}∼N\left(0,\sigma^{2}I\right)$ and is considered independent and identically distributed.

We used a sparse maximum likelihood (SML) estimator [28] adjusted for our model and data structure (S1 Text) to solve for **B** and ***μ***. SML adds an *ℓ*_1_-norm regularization term to the maximum likelihood, encouraging elements of **B** to be zero if they are not sufficiently useful to describing **Y**. A coordinate-ascent algorithm is used, allowing us to solve for the influences defined in **B** and ***μ*** on a row-by-row basis as described in Eq (2). This allows us to control which experiments are used to solve for the *i*^th^ row of **B** and ***μ***, $b_{i}^{T}$ and *μ*_*i*_ respectively. This is important because we do not want to include the experiments where component *i* was targeted. In those experiments, an outside influence that is not included in the model is impacting its abundance. Only transcripts were considered to be targets at this stage, as those are what is directly modified in the knockdown experiments. See S1 Text for more details on the model development and SML approach.

#### Estimating monolignol transcripts and proteins

We can use the influences **B** and ***μ*** solved for in the model development stage and Eq (4) to estimate how knocking down a single or combination of monolignol genes alters the abundances of the untargeted monolignol transcripts and proteins.

$$\begin{array}{c} y_{\text{pred}}={\left(I-K_{\text{targ}}B\right)}^{-1}\left(K_{\text{targ}}\mu +x_{\text{targ}}\right). \end{array}$$ (4)

We set the abundance of our targeted components to the desired knocked down amount using the vector **x**_targ_ ∈ ℜ^*M*^, and remove the model influences that would alter these set abundances using **K**_targ_ ∈ ℜ^*M*×*M*^. Where **x**_targ_ = ∑_*i*∈targ_ *x_i_***e**_*i*_ and $K_{\text{targ}}=I-\sum_{i\in \text{targ}}e_{i}^{}e_{i}^{T}$. **e**_*i*_ is the *i*^th^ unit vector. This configuration allows us to set the targeted monolignol gene components to a desired value while keeping the relationships that influence the untargeted monolignol transcripts and proteins.

A drawback of using the additive linear model to describe both the monolignol transcripts and proteins, is that a complete knockout of a targeted transcript may not result in our model estimating its protein to be completely knocked out as well. This presents an issue if the goal is to examine the impact of complete knockouts of targeted monolignol genes. To get around this issue, we assume that the targeted change in a transcript results in a proportional change to its protein abundance. For example, if we want to see what happens when we knock transcript 1, *t*_1_ down to 10% of its wildtype abundance, then $x_{\text{targ}}^{T}=[\begin{array}{ccccccc} 0.1\cdot t_{1}^{wt} & 0 & \cdots & 0.1\cdot p_{1}^{wt} & 0 & \cdots & 0 \end{array}]$ and $K_{\text{targ}}=I-e_{1}^{}e_{1}^{T}-e_{1+M/2}^{}e_{1+M/2}^{T}$.

### Differential abundance analysis

We performed the differential abundance analysis for the monolignol gene transcripts [4] using the R package DESeq2 [38] for each batch individually using the RNA-seq libraries available under GEO accession number GSE78953. The proteomics data [4] was log2 transformed and the limma package [39, 40] was used for each batch to identify significant differential abundance [41]. The proteomics data set is available on CyVerse (http://mirrors.iplantcollaborative.org/browse/iplant/home/shared/LigninSystesmDB).

### Missing data imputation

In the proteomics data set, 83 out of the 4500 proteins measured (1.8%) could not be quantified. We employed a series of rules to estimate these missing values: 1) If the protein was successfully measured for at least one other replicate in the same line, then the missing value was replaced with the average abundance of the protein from the other replicates of that line. This accounted for 42 of the missing values. 2) If a protein was not quantified for all replicates of an experimental line, then 2a) if the missing value is for a protein associated with the monolignol gene targeted for knockdown, we replaced the missing value with the fraction of its average wildtype abundance that its associated transcript was knocked down. For example, if the associated transcript was knocked down to 10% of its average wildtype value, then the missing protein value was replaced with 10% of its average wildtype value. This accounted for 30 of the missing values. 2b) The remaining missing values were replaced with the average wildtype value of that protein. This accounted for 11 of the missing values.

## Supporting information

S1 Text Supporting information. (PDF)

S1 Fig Monolignol gene transcript and protein differential abundance (cont.). (A) *PtrPAL1* knockdown experiments (Construct a1). (B) *PtrPAL2*, *PtrPAL4*, and *PtrPAL5* knockdown experiments (Construct i7). (C) *PtrPAL4* knockdown experiments (Construct a3). (D) *PtrPAL5* knockdown experiments (Construct a4). (E) *PtrPAL2* knockdown experiments (Construct a5). (F) *PtrPAL1* and *PtrPAL3* knockdown experiments (Construct i6). Gray boxes are due to missing data. Rows are the monolignol gene names, with the targeted genes for each experiment in purple. Columns are the experimental lines. * indicates p_adj_<0.05.
(TIF)

S2 Fig Monolignol gene transcript and protein differential abundance (cont.). (A) *PtrPAL1*-*PtrPAL5* knockdown experiments (Construct i8). (B) *PtrC3H3* knockdown experiments (Construct i20). (C) *PtrCAD1* knockdown experiments (Construct i33). (D) *PtrC4H2* knockdown experiments (Construct a9). (E) *PtrC4H1* knockdown experiments (Construct a10). (F) *PtrCCR2* knockdown experiments (Construct i26). Gray boxes are due to missing data. Rows are the monolignol gene names, with the targeted genes for each experiment in purple. Columns are the experimental lines. * indicates p_adj_<0.05.
(TIF)

S3 Fig Monolignol gene transcript and protein differential abundance (cont.). (A) *PtrHCT1* knockdown experiments (Construct a17). (B) *PtrHCT6* knockdown experiments (Construct a18). (C) *PtrCCoAOMT1* knockdown experiments (Construct a22). (D) *PtrCAld5H1* knockdown experiments (Construct a27). (E) *PtrCAld5H2* knockdown experiments (Construct a28). (F) *PtrHCT1* and *PtrHCT6* knockdown experiments (Construct i19). Gray boxes are due to missing data. Rows are the monolignol gene names, with the targeted genes for each experiment in purple. Columns are the experimental lines. * indicates p_adj_<0.05.
(TIF)

S4 Fig Monolignol gene transcript and protein differential abundance (cont.). (A) *Ptr4CL3* knockdown experiments (Construct a12). (B) *Ptr4CL5* knockdown experiments (Construct a13). (C) *PtrCCoAOMT1* and *PtrCCoAOMT2* knockdown experiments (Construct i24). (D) *PtrAldOMT2* knockdown experiments (Construct i30). Gray boxes are due to missing data. Rows are the monolignol gene names, with the targeted genes for each experiment in purple. Columns are the experimental lines. * indicates p_adj_<0.05.
(TIF)

S5 Fig Transcription factor expression in *PtrCAD1* and *PtrCAD2* knockdowns. Rows are the TFs identified in [12] that regulate the monolignol genes. Columns are the experimental lines. * indicates p_adj_<0.05.
(PDF)

S1 Table Table of relationships identified using SML approach. (CSV)
