# Supplementary material for: Modeling cross-regulatory influences on monolignol transcripts and proteins under single and combinatorial gene knockdowns in Populus trichocarpa
Source: PLoS Comput Biol. 2020 Apr 10;16(4):e1007197. doi: 10.1371/journal.pcbi.1007197 (PMC7147730; doi:10.1371/journal.pcbi.1007197)
Supplement: S1 Text — (PDF) [file pcbi.1007197.s001.pdf]

Supporting information

Edge detection

The method of solving for $\mathbf{B}$ and $\boldsymbol{\mu}$ closely follows the steps in [1] with a few minor adjustments to account for the differences in our model structure.

The log-likelihood function of the model defined in Eq (3) is:

$$L(\mathbf{Y}|\mathbf{B},\boldsymbol{\mu}) = \frac{-MN}{2}\log[2\pi\sigma^2] + N\log[|\mathbf{I}-\mathbf{B}|] - \frac{1}{2\sigma^2}||\mathbf{Y}-\mathbf{B}\mathbf{Y}-\boldsymbol{\mu}\mathbf{1}^T||_F^2. \tag{S1}$$

By removing terms that do not depend on $\mathbf{B}$ and adding a regularization term, the sparse maximum likelihood (SML) function becomes:

$$\hat{\mathbf{B}} = \arg\max_{\mathbf{B}} N\log[|\mathbf{I}-\mathbf{B}|] - \frac{1}{2\sigma^2}||\mathbf{Y}-\mathbf{B}\mathbf{Y}-\boldsymbol{\mu}\mathbf{1}^T||_F^2 - \lambda||\mathbf{B}||_{1,\mathbf{W}} \tag{S2}$$
$$s.t.\ B_{ij} = 0\ \forall(i,j)\in\mathcal{S}_q.$$

Where $\mathcal{S}_q$ contains the set of row and column indices of the entries of $\mathbf{B}$ constrained to 0, and $||\mathbf{B}||_{1,\mathbf{W}} := \sum_{i=1}^{M}\sum_{j=1}^{M} w_{ij}|B_{ij}|$. The weights w_{ij} are chosen as $\frac{1}{\tilde{B}_{ij}}$, where $\tilde{B}_{ij}$ is from a preliminary estimate of $\mathbf{B}$ using ridge regression (Eq (S3)). These weights are added to the regularization term to improve estimation accuracy in line with the adaptive lasso [1, 2]. Additionally, the sample variance from the ridge regression estimate is used as the estimate of σ^2 in Eq (S2).

$$\tilde{\mathbf{B}} = \arg\min_{\mathbf{B}} \frac{1}{2}||\mathbf{Y}-\mathbf{B}\mathbf{Y}-\boldsymbol{\mu}\mathbf{1}^T||_F^2 + \rho||\mathbf{B}||_F^2 \tag{S3}$$
$$s.t.\ B_{ij} = 0\ \forall(i,j)\in\mathcal{S}_q.$$

Both the ridge regression and SML estimates, $\tilde{\mathbf{B}}$ and $\hat{\mathbf{B}}$ respectively, can be solved on a row by row basis. This allows us to remove the experiments that targeted the ith component when solving for $\mathbf{b}_i^T = \begin{bmatrix} B_{i1} & \cdots & B_{iM}\end{bmatrix}$, the ith row of $\mathbf{B}$. Removing these experiments for each component prevents fitting $\mathbf{b}_i^T$ to data where an outside influence is controlling the abundance of the associated component.

Ridge Regression
For the ridge regression, this becomes

$$\tilde{\mathbf{b}}_i = \arg\min_{\mathbf{b}_i} \frac{1}{2}||\overset{i}{\mathbf{y}}{}_i^T - \mathbf{b}_i^T\overset{i}{\mathbf{Y}} - \overset{i}{\mu}_i\overset{i}{\mathbf{1}}{}^T||_2^2 + \rho||\mathbf{b}_i||_2^2 \tag{S4}$$
$$s.t.\ B_{ij} = 0\ \forall(i,j)\in\mathcal{S}_q.$$

Where the overset i implies the variables adjusted for the removed targeted experiments (e.g., $\overset{i}{\mathbf{y}}_i$ is the abundances for component i from the experiments that it was not targeted, and $\overset{i}{\mathbf{1}}{}^T$ is a $1\times\overset{i}{N}$ vector of ones, with $\overset{i}{N}$ is the number of experiments that do not target component i).

Minimizing Eq (S4) w.r.t $\overset{i}{\mu}_i$ gives

$$\overset{i}{\mu}_i = \overset{i}{\bar{y}}_i - \mathbf{b}_i^T \overset{i}{\bar{\mathbf{y}}} \tag{S5}$$

where $\overset{i}{\bar{y}}_i = \sum_{j=1}^{\overset{i}{N}} \overset{i}{\mathbf{y}}_{ij}/\overset{i}{N}$ and $\overset{i}{\bar{\mathbf{y}}} = \sum_{j=1}^{\overset{i}{N}} \overset{i}{\mathbf{Y}}_j/\overset{i}{N}$. Eq (S4) then becomes

$$\tilde{\mathbf{b}}_i = \arg\min_{\mathbf{b}_i} \frac{1}{2}||\overset{i}{\tilde{\mathbf{y}}}_i^T - \mathbf{b}_i^T \overset{i}{\tilde{\mathbf{Y}}}||_2^2 + \rho||\mathbf{b}_i||_2^2. \tag{S6}$$
$$s.t.\ B_{ij} = 0\,\forall(i,j) \in \mathcal{S}_q.$$

where $\overset{i}{\tilde{\mathbf{y}}}_i^T = \overset{i}{\mathbf{y}}_i^T - \overset{i}{\bar{y}}_i \overset{i}{\mathbf{1}}^T$ and $\overset{i}{\tilde{\mathbf{Y}}} = \overset{i}{\mathbf{Y}} - \overset{i}{\bar{\mathbf{y}}}\overset{i}{\mathbf{1}}^T$.

The constraint $B_{ij} = 0\,\forall(i,j) \in \mathcal{S}_q$ can be applied directly by removing the jth element(s) from $\mathbf{b}_i^T$ and the jth row(s) from $\overset{i}{\tilde{\mathbf{Y}}}$. Let $\check{\mathbf{b}}_i^T$ and $\overset{i}{\check{\mathbf{Y}}}$ represent these adjustments. Then Eq (S6) becomes

$$\tilde{\check{\mathbf{b}}}_i = \arg\min_{\check{\mathbf{b}}_i} \frac{1}{2}||\overset{i}{\tilde{\mathbf{y}}}_i^T - \check{\mathbf{b}}_i^T \overset{i}{\check{\mathbf{Y}}}||_2^2 + \rho||\check{\mathbf{b}}_i||_2^2. \tag{S7}$$

This has a close form solution

$$\tilde{\check{\mathbf{b}}}_i = \big(\overset{i}{\check{\mathbf{Y}}}\overset{i}{\check{\mathbf{Y}}}^T + \rho\mathbf{I}\big)^{-1}\big(\overset{i}{\check{\mathbf{Y}}}\overset{i}{\tilde{\mathbf{y}}}_i\big) \tag{S8}$$

Sparse Maximum Likelihood

Using the cyclic block-coordinate ascent iteration algorithm from [1], we define the matrix $\hat{\mathbf{B}}(B_{ij})$ to be a matrix equal to $\hat{\mathbf{B}}$ except for the (i,j)th entry which is replace by B_{ij}, such that $\hat{\mathbf{B}}(B_{ij}) := \hat{\mathbf{B}} + \mathbf{e}_i\mathbf{e}_j^T(B_{ij} - \hat{B}_{ij})$. Eq (S2) can then be expressed as

$$B_{ij} = \arg\max_{B_{ij}} \overset{i}{N}\sigma^2 \log[|\mathbf{I} - \hat{\mathbf{B}}(B_{ij})|] - \frac{1}{2}||\overset{i}{\mathbf{Y}} - \hat{\mathbf{B}}(B_{ij})\overset{i}{\mathbf{Y}} - \overset{i}{\boldsymbol{\mu}}\overset{i}{\mathbf{1}}^T||_F^2 - \lambda w_{ij}|B_{ij}| \tag{S9}$$

Similar to the ridge regression, maximizing Eq (S9) w.r.t $\overset{i}{\boldsymbol{\mu}}$ gives

$$\overset{i}{\boldsymbol{\mu}} = \big(\mathbf{I} - \hat{\mathbf{B}}(B_{ij})\big)\overset{i}{\bar{\mathbf{y}}} \tag{S10}$$

Substituting into Eq (S9),

$$B_{ij} = \arg\max_{B_{ij}} \overset{i}{N}\sigma^2 \log[|\mathbf{I} - \hat{\mathbf{B}}(B_{ij})|] - \frac{1}{2}||\overset{i}{\tilde{\mathbf{Y}}} - \hat{\mathbf{B}}(B_{ij})\overset{i}{\tilde{\mathbf{Y}}}||_F^2 - \lambda w_{ij}|B_{ij}| \tag{S11}$$

Following the steps from [1], by rearranging and removing constant terms, this becomes

$$B_{ij} = \arg\max_{B_{ij}} \overset{i}{N}\sigma^2 \log[|\alpha_0 - c_{ij}B_{ij}|] + \alpha_1 B_{ij} - \frac{1}{2}\alpha_2 B_{ij}^2 - \lambda w_{ij}|B_{ij}| \tag{S12}$$

where c_{ij} represents the (i,j)th co-factor of the matrix $\mathbf{I} - \hat{\mathbf{B}}$, and

$$\alpha_0 := |\mathbf{I} - \hat{\mathbf{B}}|$$

$$\alpha_1 := \left[\left(\mathbf{I} - \hat{\mathbf{B}} + \mathbf{e}_i\mathbf{e}_j^T\hat{B}_{ij}\right)\overset{i}{\tilde{\mathbf{Y}}}\overset{i}{\tilde{\mathbf{Y}}}^T\right]_{ij}$$

$$\alpha_2 := ||\overset{i}{\tilde{\mathbf{Y}}}^T\mathbf{e}_j||_2^2$$

The value of B_{ij} can then be solved for as described in equations (12)-(16) and the associated text in [1].

Regularization parameters

The regularization parameters ρ and λ, were found using 5-fold cross-validation while stepping through a set range of values for ρ and λ in the ridge regression and SML algorithms respectively. To improve stability, the cross-validations were run multiple times and the ρ and λ resulting in the lowest mean error across the testing folds were selected and used to solve for $\tilde{\mathbf{B}}$ and $\hat{\mathbf{B}}$ in Eq (S3) and Eq (S2).

Algorithm implementation

The Matlab code for the SML algorithm provided by [1] was modified based on the changes described above. Additionally, the abundances of the monolignol transcripts and proteins ranged from on the order of 10^{-1} nM and 10^4 nM. For this reason, we included a function to scale each transcript and protein by its maximum value in the training data to bring the range of each transcript and protein to between 0 and 1 when solving for $\tilde{\mathbf{B}}$ and $\hat{\mathbf{B}}$. The coefficients of the returned $\tilde{\mathbf{B}}$ and $\hat{\mathbf{B}}$ were then accordingly untransformed to work with un-scaled data.

References

[1] Cai X, Bazerque JA, Giannakis GB. Inference of Gene Regulatory Networks with Sparse Structural Equation Models Exploiting Genetic Perturbations. PLoS Computational Biology. 2013;9(5):e1003068. doi:10.1371/journal.pcbi.1003068.

[2] Zou H. The Adaptive Lasso and Its Oracle Properties. Journal of the American Statistical Association. 2006;101(476):1418–1429.
