# Supplementary figures and images for: Modeling cross-regulatory influences on monolignol transcripts and proteins under single and combinatorial gene knockdowns in Populus trichocarpa
Source: PLoS Comput Biol. 2020 Apr 10;16(4):e1007197. doi: 10.1371/journal.pcbi.1007197 (PMC7147730; doi:10.1371/journal.pcbi.1007197)

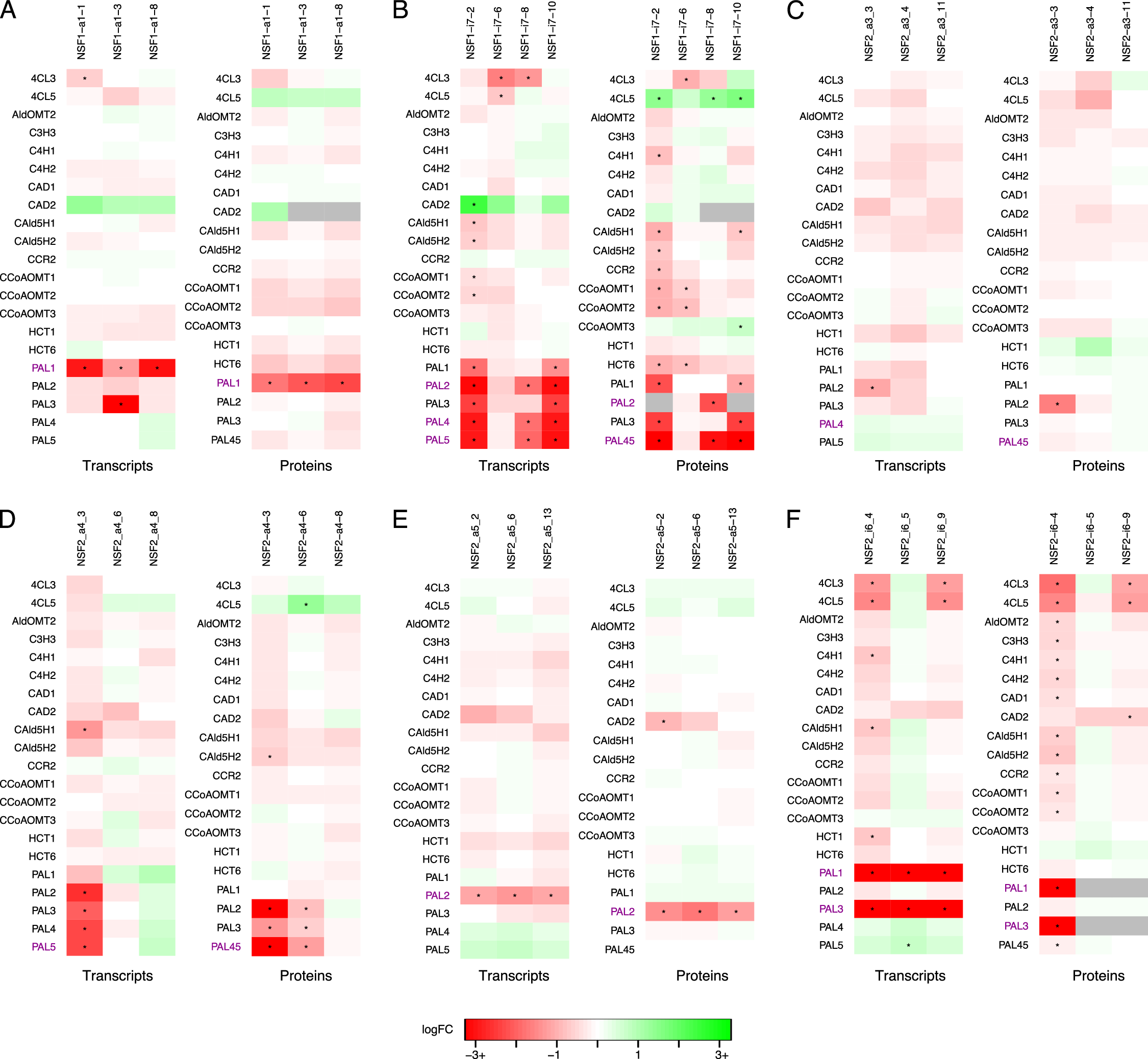

Supplement: S1 Fig — (A) PtrPAL1 knockdown experiments (Construct a1). (B) PtrPAL2, PtrPAL4, and PtrPAL5 knockdown experiments (Construct i7). (C) PtrPAL4 knockdown experiments (Construct a3). (D) PtrPAL5 knockdown experiments (Construct a4). (E) PtrPAL2 knockdown experiments (Construct a5). (F) PtrPAL1 and PtrPAL3 knockdown experiments (Construct i6). Gray boxes are due to missing data. Rows are the monolignol gene names, with the targeted genes for each experiment in purple. Columns are the experimental lines. * indicates padj<0.05. (TIF) [file pcbi.1007197.s002.tif]

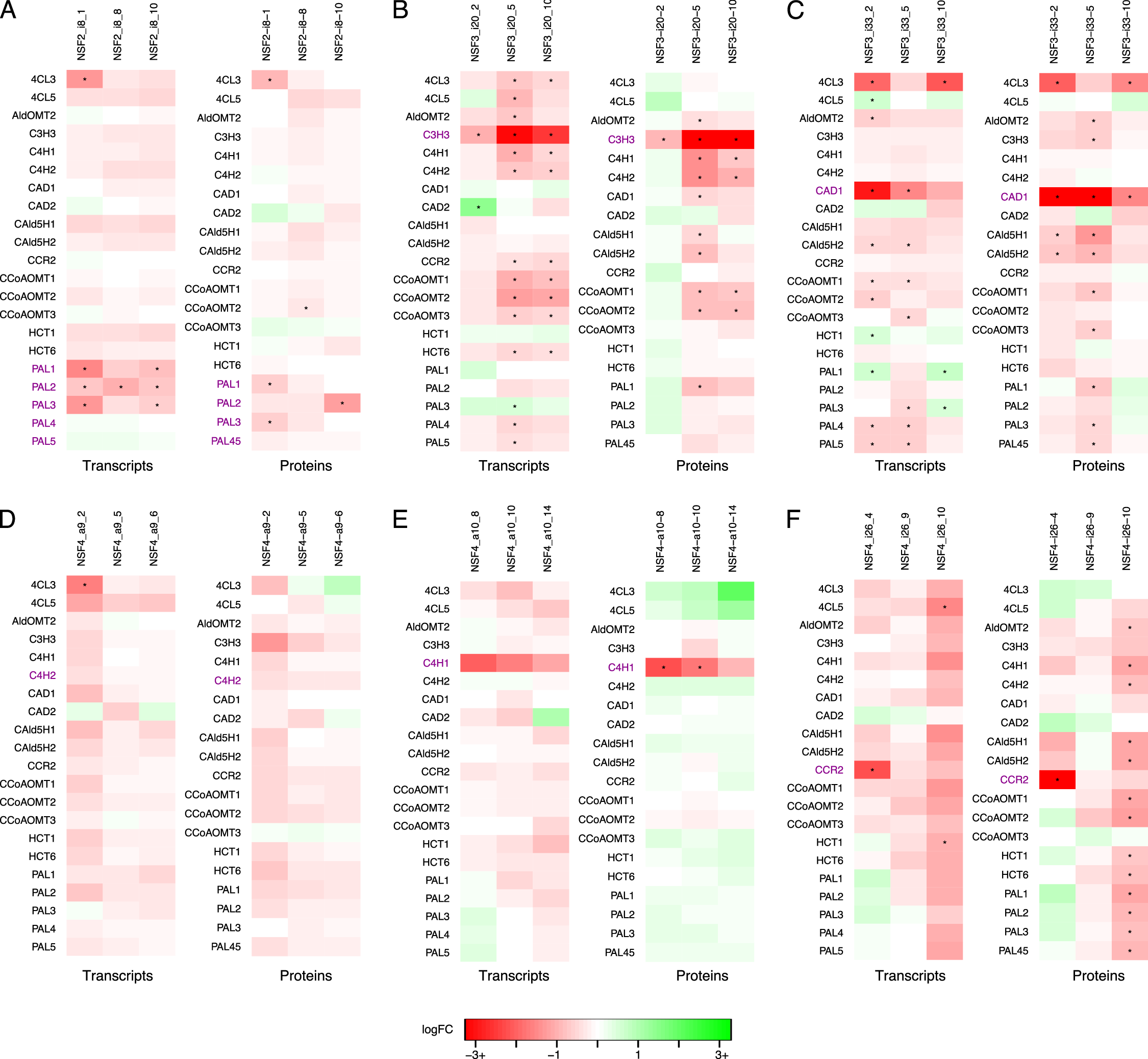

Supplement: S2 Fig — (A) PtrPAL1-PtrPAL5 knockdown experiments (Construct i8). (B) PtrC3H3 knockdown experiments (Construct i20). (C) PtrCAD1 knockdown experiments (Construct i33). (D) PtrC4H2 knockdown experiments (Construct a9). (E) PtrC4H1 knockdown experiments (Construct a10). (F) PtrCCR2 knockdown experiments (Construct i26). Gray boxes are due to missing data. Rows are the monolignol gene names, with the targeted genes for each experiment in purple. Columns are the experimental lines. * indicates padj<0.05. (TIF) [file pcbi.1007197.s003.tif]

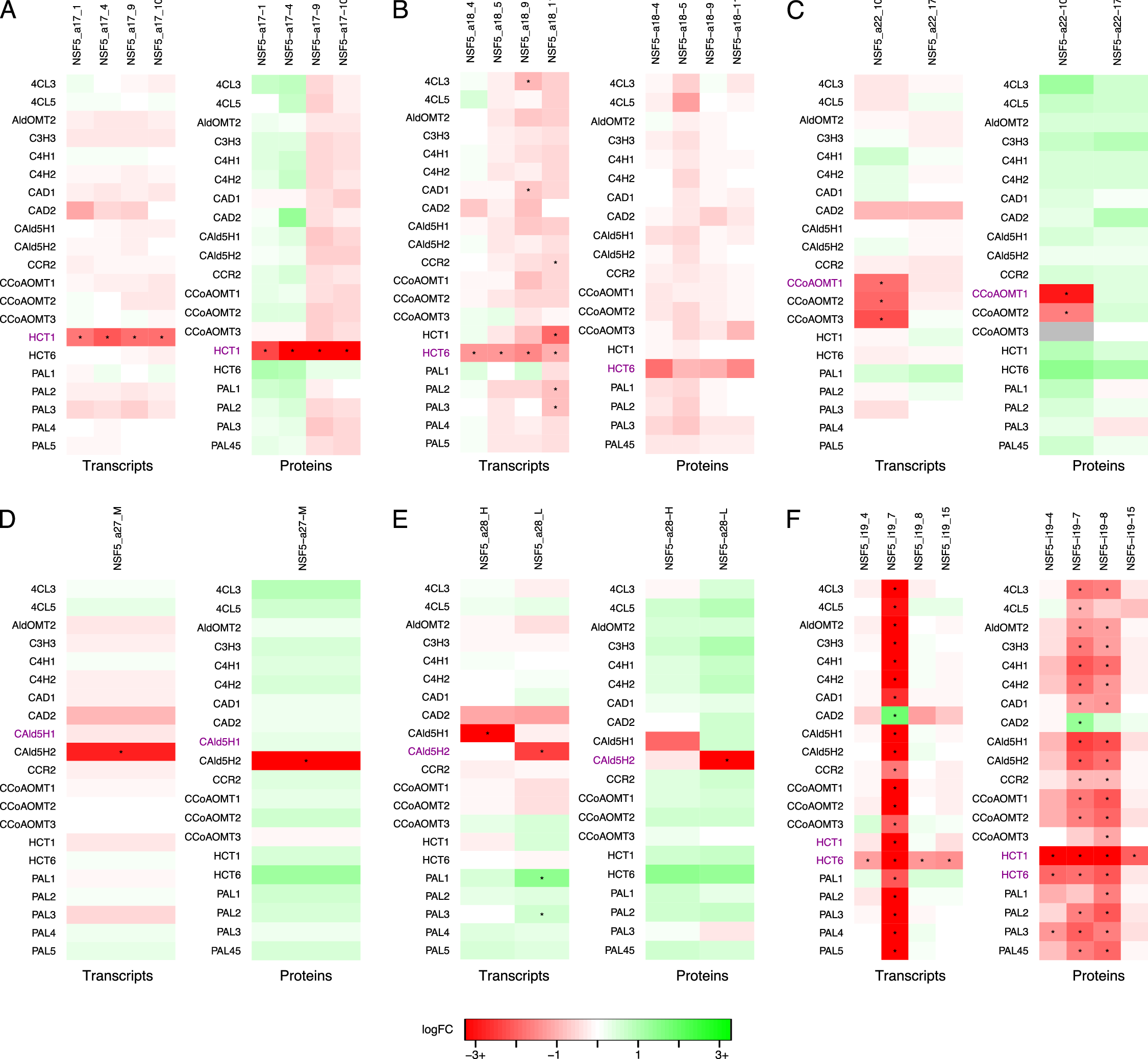

Supplement: S3 Fig — (A) PtrHCT1 knockdown experiments (Construct a17). (B) PtrHCT6 knockdown experiments (Construct a18). (C) PtrCCoAOMT1 knockdown experiments (Construct a22). (D) PtrCAld5H1 knockdown experiments (Construct a27). (E) PtrCAld5H2 knockdown experiments (Construct a28). (F) PtrHCT1 and PtrHCT6 knockdown experiments (Construct i19). Gray boxes are due to missing data. Rows are the monolignol gene names, with the targeted genes for each experiment in purple. Columns are the experimental lines. * indicates padj<0.05. (TIF) [file pcbi.1007197.s004.tif]

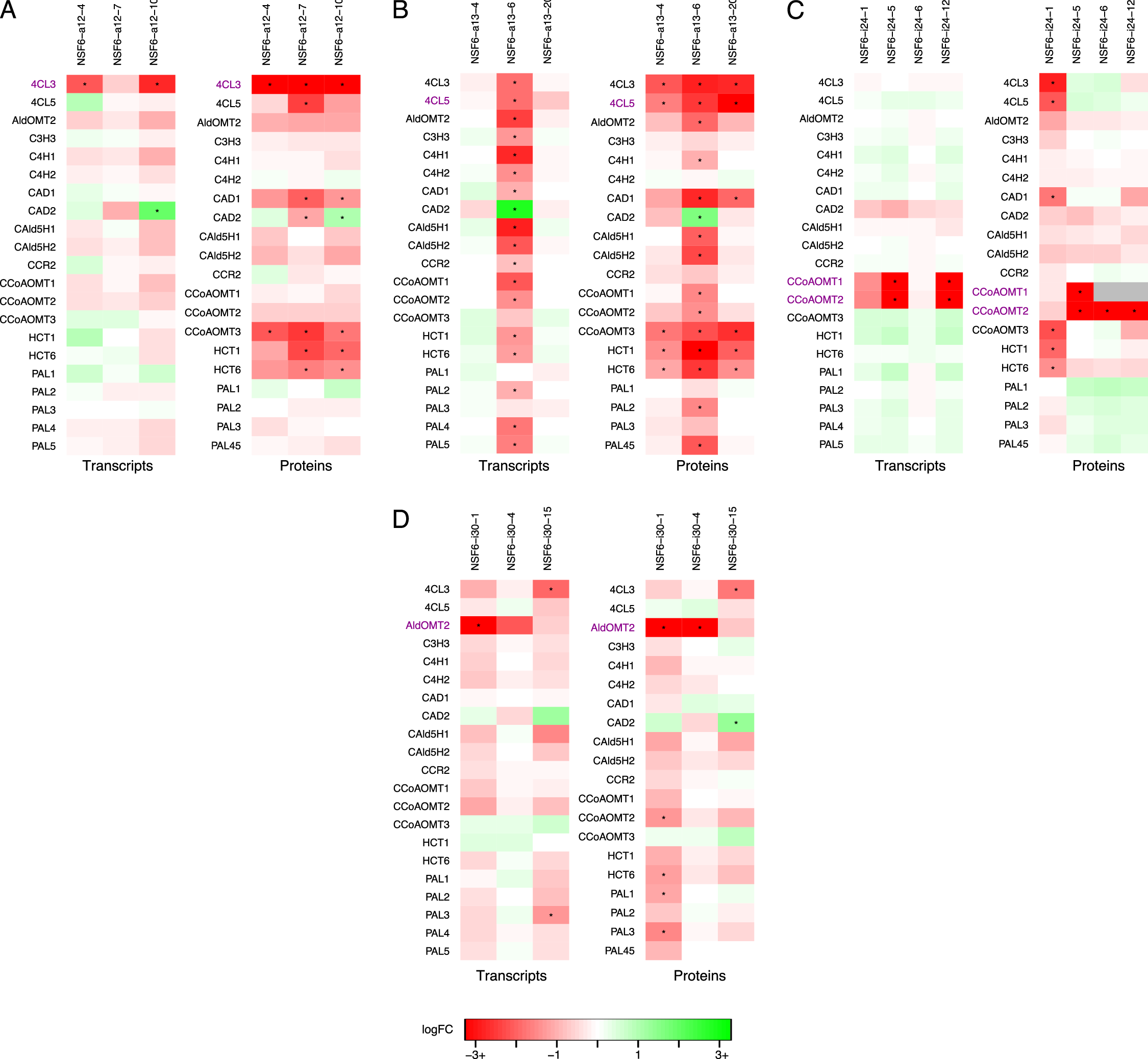

Supplement: S4 Fig — (A) Ptr4CL3 knockdown experiments (Construct a12). (B) Ptr4CL5 knockdown experiments (Construct a13). (C) PtrCCoAOMT1 and PtrCCoAOMT2 knockdown experiments (Construct i24). (D) PtrAldOMT2 knockdown experiments (Construct i30). Gray boxes are due to missing data. Rows are the monolignol gene names, with the targeted genes for each experiment in purple. Columns are the experimental lines. * indicates padj<0.05. (TIF) [file pcbi.1007197.s005.tif]

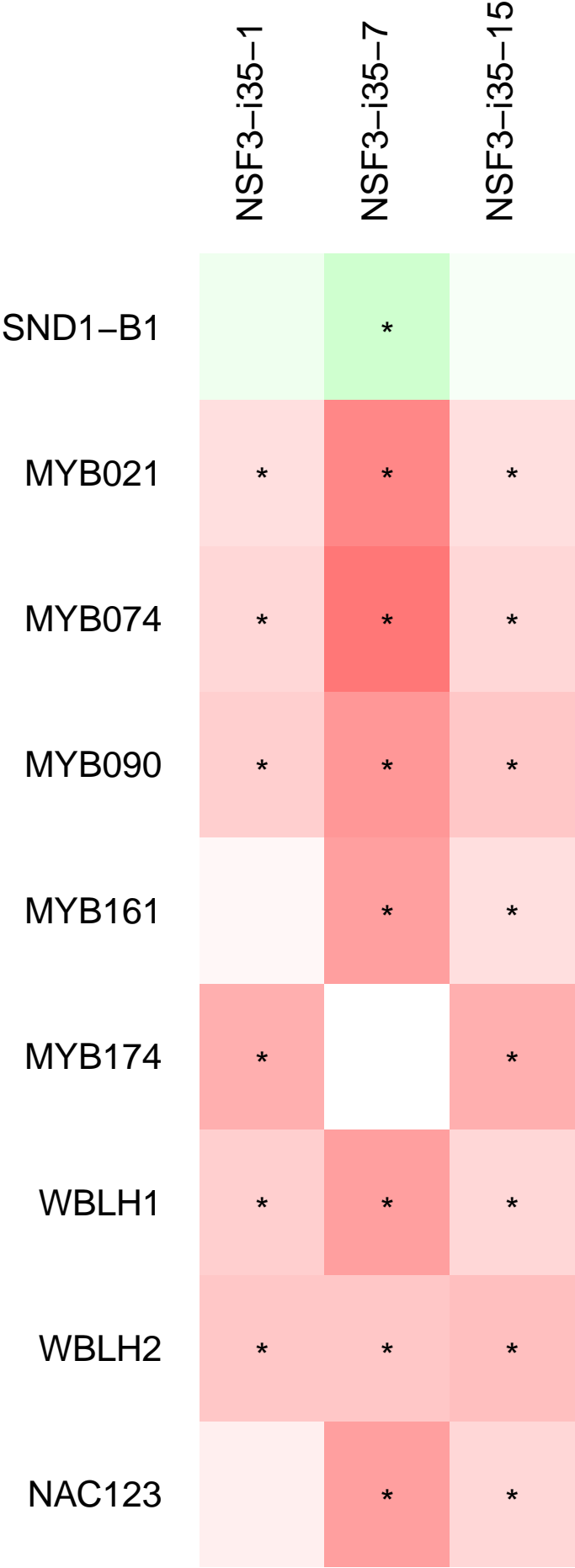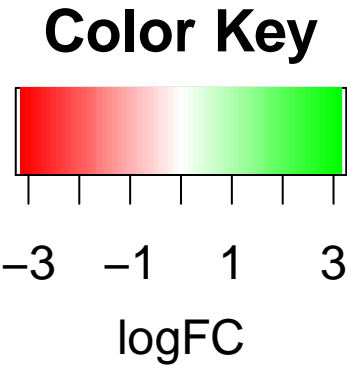

Supplement: S5 Fig — Rows are the TFs identified in [12] that regulate the monolignol genes. Columns are the experimental lines. * indicates padj<0.05. (PDF) [file pcbi.1007197.s006.pdf]
